# Extracellular Vesicles as Biomarkers and Non-Surgical Therapeutics in Cardiovascular Diseases

**DOI:** 10.3390/jcm15041537

**Published:** 2026-02-15

**Authors:** Dana A. Almazroua, Kelsey C. Muir, M. Ruhul Abid

**Affiliations:** 1 College of Medicine, Alfaisal University, Riyadh 11533, Saudi Arabia; 2 Cardiovascular Research Center, Department of Surgery, Rhode Island Hospital, The Warren Alpert Medical School of Brown University, Providence, RI 02903, USA

**Keywords:** extracellular vesicles, myocardial ischemia, bioengineering, stem cells

## Abstract

**Background**: Cardiovascular disease (CVD), including myocardial ischemia, remains the leading cause of mortality. Current therapies for ischemic myocardium rely largely on invasive revascularization strategies, highlighting the need for improved non-invasive diagnostic and therapeutic approaches. Recent studies suggest that extracellular vesicles (EVs) play a critical role in cardiovascular pathophysiology and may offer novel clinical applications. **Methods**: This review synthesizes current preclinical and clinical literature on EV biology, including their classification, isolation, and characterization methods, and mechanisms of Intercellular communication. Published studies evaluating EVs as biomarkers and non-surgical therapeutics across major cardiovascular conditions were critically analyzed. **Results**: EVs facilitate intercellular communication by transferring bioactive molecules that influence disease progression and cardiac repair. Accumulating evidence supports their potential utility as biomarkers for disease prediction and severity assessment, as well as cell-free therapeutics in myocardial infarction, cardiomyopathies, atrial fibrillation, and heart failure. However, significant gaps remain, including the lack of validated EV-based biomarkers, inconsistent isolation and characterization methodologies, limited in vivo tracking data, and barriers to clinical translation. **Conclusions**: EVs represent a promising frontier in non-invasive cardiovascular diagnostics and therapeutics. Addressing current methodological and translational challenges, alongside advances in EV bioengineering, will be essential to realize their full clinical potential in CVD management.

## 1. Introduction

Cardiovascular diseases (CVDs) pose a significant burden on society, and despite medical advancements, challenges remain in treating these severe, life-threatening conditions. According to the American Heart Association (AHA), approximately 19.1 million deaths worldwide were attributed to CVDs. Additionally, the economic burden in the United States is substantial, with costs around USD 219 billion annually. Current treatment modalities include lifestyle modifications, such as a healthy diet and exercise, guideline-directed medical therapy including angiotensin-converting enzyme (ACE) inhibitors, β-blockers, antiplatelet therapy (e.g., aspirin), statins, calcium channel blockers (CCBs), diuretics, and surgical interventions such as coronary artery bypass grafting (CABG), percutaneous coronary interventions (PCI), and valve replacement. However, these approaches are sometimes inadequate for treating certain conditions, such as specific cases of heart failure. Some heart failure patients are not suitable candidates for lifesaving invasive surgical procedures due to factors such as age, comorbidities, severity of their disease, or prior insufficient revascularization. Therefore, exploring diverse treatment options that accommodate these patients’ needs is essential. We begin by exploring an overview of stem cell therapy, including the limitations in clinical translatability. Ultimately, we will focus on a promising non-surgical therapeutic approach currently being studied: the administration of extracellular vesicles (EVs) in CVD with a major focus on ischemic heart disease (IHD), as well as EV types/classes, isolation methods, mechanism of action, advantages, limitations, bioengineering opportunities for directed delivery, and future directions. Given the limitations in the non-surgical treatment of CVD, there is a rising need for innovative therapeutic approaches. One such is the use of stem cell-derived EV therapy.

## 2. Stem Cell Therapy

One of the emerging modalities for treating CVDs is stem cell therapy. Various types of stem cells have been used in this context, including embryonic stem cells (ESCs), adult stem cells (ASCs), cardiac stem cells (CSCs), and induced pluripotent stem cells (iPSCs) [1]. Among these, mesenchymal stem cells (MSCs) are the leading candidates for cellular therapy due to their multipotent nature, non-hematopoietic origin, and capability of self-renewal and differentiation. However, despite robust differentiation potential in vitro, MSCs display highly limited differentiation in vivo, with poor engraftment rates and an inability to generate non-mesenchymal lineages [2,3,4,5].

MSCs primarily exert their therapeutic effects through trophic support mechanisms, including immunomodulation and the secretion of bioactive molecules that promote tissue repair [6,7,8,9]. MSCs derived from bone marrow, adipose tissue, and umbilical cord display distinct characteristics (Table 1), yet share similar limitations in vivo [10].

This growing recognition has driven interest in identifying the specific components within MSC-CM that are responsible for these beneficial effects. Among these components, extracellular vesicles (EVs)—including small-EVs and microvesicles—have emerged as key mediators of MSC-induced paracrine effects [11]. EVs carry a diverse cargo of proteins, lipids, and nucleic acids that can influence recipient cells and modulate cellular processes essential for tissue repair and regeneration. As a result, EV-based therapies are now being explored as a cell-free alternative to MSC transplantation, offering potential advantages in terms of stability, scalability, and reduced risk of immune rejection. Accordingly, EVs are increasingly viewed as a cell-free strategy that addresses key limitations of stem cell transplantation, including safety and scalability.

**Table 1:** Comparison of mesenchymal stem cells (MSCs) derived from bone marrow (BMSCs), adipose tissue (ADSCs), and umbilical cord (UC-MSCs) with respect to harvesting procedure, age-related effects on cell quantity and quality, renewal capacity, and expression of embryonic markers.

Characteristics	BMSCs	ADSCs	UC-MSCs	References
**Harvesting procedure**	Invasive	Invasive	Non-invasive	[10,12]
**Effect of age on cell quantity and quality**	Declines with donor age	Declines with donor age	Unaffected	[10,12]
**Cell renewal capabilities**	Lower	Lower	Higher	[10,12]
**Expression of embryonic markers**	Lower	Lower	Higher	[10,12]

## 3. Extracellular Vesicles

Extracellular vesicles (EVs) are membrane-bound vesicles released from almost all cells in the body into the extracellular space [13,14]. EVs act as communicating agents between adjacent and distant cells by releasing their cargo, which includes various proteins, lipids, messenger ribonucleic acids (mRNAs), microRNAs, DNAs, cytokines, growth factors, enzymes, and organelles [15,16]. Since the discovery of EVs, extensive research has been conducted to understand their biology, function, and possible therapeutic applications. Researchers are intrigued by the potential of EVs in gene and drug delivery, disease diagnosis, and biomarker-driven therapy [17].

The importance of EVs lies in their ability to deliver information to recipient cells, thereby influencing their microenvironmental milieu and function. The unique cargo determines its effects on the recipient cells, whereby the surface receptors and ligands determine its biodistribution and capacity to bind to target cells. The predominant mechanism for the recipient cells to uptake the EVs is phagocytosis. Once internalized, EVs integrate their lipids, proteins, and genetic information into the recipient cells, causing alterations in their physiological or pathological state [18].

### 3.1. Classification of EVs

EVs are divided based on their sizes, content, mode of secretion, and function into three main categories: small EVs (often referred to as exosomes), microvesicles, and apoptotic bodies. Small EVs range in size from 40 to 150 nm and are released by the fusion of the multivesicular bodies within the plasma membrane. Microvesicles range in size from 150 nm to 1 µm and are formed through budding from the cell membrane. Apoptotic bodies range in size from 1 to 5 µm and are shed from apoptotic cells through membrane blebbing.

Furthermore, specific biomarkers characterize EVs, in which commonly found markers include the tetraspanin (TET) family of proteins (e.g., CD9, CD37, CD63, CD81, CD82), ALIX, TSG101, and integrins. Mass spectrometry has identified additional abundant proteins in EVs, such as CD47, CD29 (also known as ITGB1), ATP1A1, SLC1A5, SLC3A2, and BSG [19,20,21,22]. A study was conducted to quantify the biomarkers present on the surface of EVs and assess the variability of their expression, confirming the most abundantly found markers as above. Other markers were present in less than 20% of the EVs.

**Figure 1:**
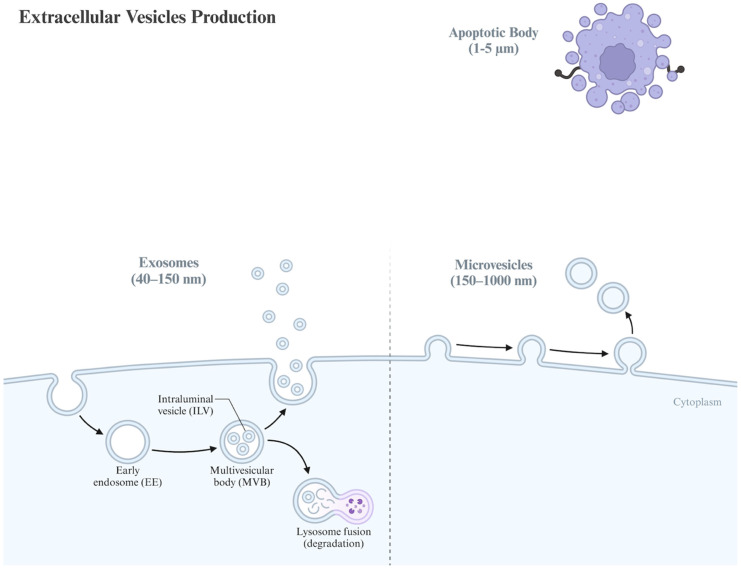
Depicting the mechanisms of small EVs (exosomes) and microvesicle production. Exosomes are formed within multivesicular bodies (MVBs), which can fuse with either the plasma membrane to release small EVs or with lysosomes for degradation. Microvesicles are generated through the direct outward budding of the plasma membrane. Created with BioRender.com.

Each category of EVs carries a distinct set of microRNAs (miRNAs), small interfering RNAs (siRNAs), proteins, lipids, cytokines, and growth factors [23,24]. The International Society for Extracellular Vesicles (ISEV) has proposed more specific criteria for classifying EVs due to the overlap in their sizes and the complexity of their recognition. They recommend characterizing EVs using a combination of techniques, including nanoparticle tracking analysis (NTA), flow cytometry, transmission electron microscopy, and Western blot [25]. In the subsequent sections, we discuss in detail each type of EV. A schematic overview of the structure, biogenesis, and release pathways of the different EV subtypes is illustrated in (Figure 1).

#### 3.1.1. Small EVs

Small EVs (often referred to as exosomes) are EVs formed from endosomes through a three-step mechanism: biogenesis, transport, and release [26]. Small EVs are released ubiquitously and have been found in plasma, urine, semen, saliva, bronchial fluid, cerebrospinal fluid (CSF), breast milk, serum, amniotic fluid, synovial fluid, tears, lymph, and bile [27,28,29,30,31,32,33,34,35].

For the biogenesis of small EVs, proteins are transferred into early endosomes, followed by the maturation process, whereby they develop into late endosomes and multivesicular bodies (MVBs). This maturation process involves the invagination of the endosomal membrane, resulting in the formation of intraluminal vesicles (ILVs) within the MVBs. MVBs eventually have two primary outcomes: they can be degraded by lysosomes with all their contents, or they can fuse with the cell membrane and be eventually extruded. This fusion process, facilitated by the lipid ceramide, causes the release of the ILVs as small EVs and influences the surrounding microenvironment via paracrine signaling [36,37,38].

Researchers have suggested that the formation of MVBs is influenced by growth factors, which allows the cells to adjust their exosome production according to their needs. Consequently, this set of proteins is termed “exosomal marker proteins” [39]. The formation of small EVs and the transportation of MVBs are regulated by the endosomal sorting complexes required for transport (ESCRT) pathway [40]. Subsequently, small EVs are characterized by ESCRT proteins (Alix, TSG101, HSC70, and HSP90β) and tetraspanins (CD63, CD81, and CD9), which serve as key surface markers found in higher concentrations compared to the overall cell lysate, independent of their cellular origin, and remain detectable even in ESCRT-independent exosome release mechanisms [41,42,43,44,45]. The determination of the fate of a specific MVB is not entirely understood [46]. However, some studies have indicated that the amount of cholesterol present within a particular MVB can influence its fate. A study reported that in morphologically identical MVBs, those rich in cholesterol were secreted, whereas those lacking cholesterol were sent to lysosomes for degradation [46].

#### 3.1.2. Microvesicles

Microvesicles form through the direct outward budding of the cell’s plasma membrane. Although the precise mechanistic steps of microvesicle formation are not completely understood, it is believed to require cytoskeleton components, such as actin and microtubules, motor molecules including kinesins and myosins, and ultimately, the fusion machinery of SNARES and tethering factors. Given that microvesicles form by outward budding of the cell’s plasma membrane, it is logical that they primarily contain cytosolic and plasma membrane-associated proteins, particularly those known to aggregate at the plasma membrane surface, such as tetraspanins. Other commonly found proteins in microvesicles include cytoskeletal proteins, heat shock proteins, integrins, and proteins with post-translational modifications like glycosylation and phosphorylation. It is not surprising that proteins associated with certain organelles, such as the mitochondria or Golgi apparatus, are typically absent in microvesicles. This key difference in locational protein differentiation enables easier characterization of microvesicles from small EVs [47,48].

#### 3.1.3. Apoptotic Bodies

Apoptotic bodies are expelled by dying cells into the extracellular space [49]. During apoptosis, the cell undergoes contraction, which increases the hydrostatic pressure within the cell, leading to detachment of the plasma membrane from the cytoskeleton and subsequent formation of apoptotic bodies. Given their formation mechanism, it is reasonable that apoptotic bodies, unlike small EVs and microvesicles, contain intact organelles, chromatin, and glycosylated proteins [50,51]. Therefore, proteins associated with the nucleus (i.e., histones), mitochondria (i.e., HSP60), Golgi apparatus, and endoplasmic reticulum (i.e., GRP78) are found at higher levels in apoptotic bodies compared to small EVs and microvesicles. Moreover, the proteomic profile of apoptotic bodies closely resembles that of the cell lysate, whereas significant differences exist between the proteomic profiles of small EVs and the cell lysate. 

## 4. Isolation of EVs

A major challenge in the therapeutic use of EVs is the heterogeneity of the methods used to isolate and purify their different populations, highlighting the need for standardized protocols. Various isolation methods have been employed, including differential ultracentrifugation, size-exclusion chromatography, ultrafiltration, immunocapture, and microfluidics [52,53,54]. The gold standard method for isolating EVs from plasma, tissues, and cells is ultracentrifugation [55,56,57]. Table 2 summarizes the most common isolation methods and their respective advantages and disadvantages.

**Table 2:** Comparative summary of extracellular vesicle (EV) isolation methods, outlining their respective advantages, limitations, and technical considerations.

Method of Isolation	Advantages	Disadvantages	References
**Ultracentrifugation**	Higher number of EVs isolates, cost, isolation from large volumes, no additional chemicals, simple, adaptable	Time consuming, co-isolation of contaminants, equipment, low reproducibility, exosomal damage, co-pelleting	[58,59]
**Density Gradient Centrifugation**	High purity, no additional reagents	Complex procedure, loss of samples	[59]
**Ultrafiltration**	Simple procedure, pure preparations, no limitations on sample volume, simultaneous processing of multiple samples	Loss of samples, contamination, poor biological activity	[58,59]
**Size-exclusion Chromatography**	Improved speed	Co-purification of proteins, matrix/membrane interactions	[60]
**Microfluidic Devices**	High purity, efficiency, less samples required	Complexity of devices, need for additional equipment, inconsistency across protocols, often only suitable at small scale	[60]
**Precipitation**	Rapid, lower cost	Co-precipitation is almost unavoidable, inconsistent	[60]

Different EV isolation techniques can significantly influence the composition, purity, and downstream interpretation of EVs. Classical differential ultracentrifugation (dUC) is widely used but often co-isolates non-vesicular proteins and produces heterogeneous EV populations, which can bias proteomic and functional analyses. In contrast, size-exclusion chromatography (SEC) yields EVs with lower contaminating protein content and better preservation of vesicle structure, improving reproducibility and functional interpretation across studies [61]. Methods such as polymer precipitation and commercial kits may increase yield, but often at the cost of co-precipitating soluble proteins and lipoproteins, potentially confounding analyses of EV cargo such as RNA or protein content [62]. Comparative studies have shown that the choice of isolation method alters measured miRNA and proteomic profiles, underscoring that EV composition and subsequent interpretations depend on the isolation strategy employed [63]. Furthermore, combined techniques (e.g., ultrafiltration + SEC) can enhance purity and reproducibility, highlighting that methodological selection impacts both the biological characteristics of isolated EVs and the robustness of downstream findings [64].

## 5. EVs in Cardiovascular Diseases

**Figure 2:**
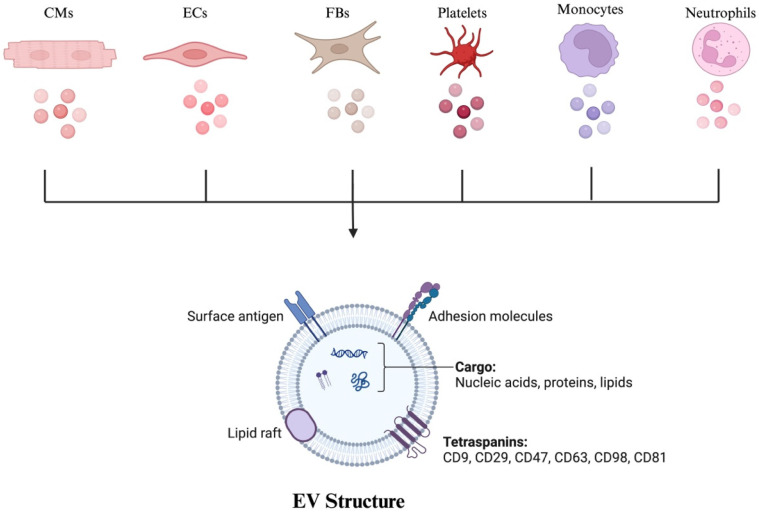
Illustrating various cellular sources of extracellular vesicles (EVs) within the cardiovascular system, including cardiomyocytes (CMs), endothelial cells (ECs), fibroblasts (FBs), platelets, monocytes, and leukocytes. Additionally, it depicts the structural components of an extracellular vesicle (EV). Created with BioRender.com.

Regarding the cardiovascular system, studies have indicated that EVs can be released from a multitude of cell types, including cardiomyocytes (CMs), fibroblasts (FBs), endothelial cells (ECs), platelets, leukocytes, monocytes, and macrophages (Figure 2). Under physiological conditions, EVs play an important role in cell-to-cell communication, maintaining normal cardiac structure and function [65]. However, under pathological conditions, EVs change their composition and contribute to the development of CVDs [66]. For instance, EVs can initiate lesion formation in atherosclerosis, promote calcification and plaque formation, and trigger thrombus formation following plaque rupture [67]. Conversely, EVs derived from stem cells in vitro can protect against the development of cardiovascular diseases and promote cardiac repair. Therefore, EVs have great potential in monitoring various CVDs, in which we overview their potential as biomarkers. Furthermore, we focus on the roles of EVs in common disease states, including myocardial infarction, cardiomyopathies, atrial fibrillation, and heart failure, as well as their therapeutic potential in each disease type. Recent studies have also highlighted the therapeutic and diagnostic relevance of extracellular vesicles across multiple CVD contexts [68].

### 5.1. EVs as Biomarkers in Cardiovascular Diseases

The use of biomarkers in various CVDs has been extensively researched over the past three decades, leading to the development of highly sensitive screening tests. For example, cardiac troponin (with high-sensitivity assays) plays a crucial role in early myocardial infarction (MI) diagnosis, while inflammatory markers like high-sensitivity C-reactive protein (CRP) help predict MI and mortality [69]. Advancements in the understanding of biomarkers associated with various pathological conditions facilitate the early detection and diagnosis of a wide range of cardiovascular disorders [70]. However, the prognostication on patient outcomes cannot be extrapolated from such biomarkers.

Therefore, the investigation into EVs as biomarkers has become an attractive solution. The count, content, and origin of EVs can provide critical information about the phenotype of their cells of origin, thereby elucidating the physiological or pathological state of these cells. EV determination is enabled by using flow cytometry with size calibration and optics suitable for small molecule detection [71]. The combined measurement of small particles along with the specific surface markers provides accurate extraction of EVs.

Circulating levels of EVs have been shown to increase in various CVDs, serving as important diagnostic and prognostic markers [72]. For example, in conditions of increased platelet activation, such as myocardial infarction, the release of platelet-derived EVs is elevated, and patients with atherosclerosis have shown increased levels of leukocyte-derived EVs in their plasma, which have been associated with disease progression [73,74]. A prospective study demonstrated higher levels of circulating endothelial cell-derived EVs in patients with heart failure compared to healthy subjects [75].

**Figure 3:**
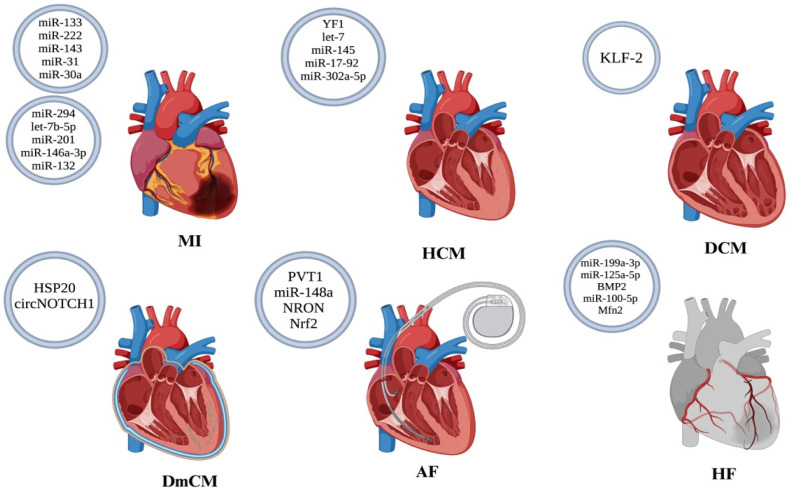
Demonstrating various microRNAs (miRNAs) encapsulated within extracellular vesicles (EVs) that exhibit potential therapeutic applications in myocardial infarction (MI), hypertrophic cardiomyopathy (HCM), dilated cardiomyopathy (DCM), diabetic cardiomyopathy (DmCM), atrial fibrillation (AF), and heart failure (HF). Created with BioRender.com.

In regard to patient prognosis, once diagnosed, EVs may play a telling role. Non-coding RNAs, particularly microRNAs (miRNAs), have been implicated as biomarkers and therapeutic molecules in myocardial infarction (MI) and other cardiovascular diseases [72] (Figure 3). For example, in CAD, increased expression of miRNA-126 in circulating EVs is associated with a reduced risk of major cardiovascular outcomes. Research has also demonstrated a negative correlation between the circulating levels of EV-miRNA-126 and levels of Cardiac Troponin I (cTnI), a marker for MI, and N-terminal pro-B-type Natriuretic Peptide (NT-proBNP), a marker for heart failure, suggesting that miRNA-126 can serve as a useful positive biomarker after acute events [76].

Given the important role of EVs in CVDs, recent research has also investigated their function across different disease states, their molecular cargo, and their contribution to disease pathogenesis. Understanding these roles provides valuable insights into their potential as biomarkers and therapeutic agents in CVDs.

### 5.2. EVs in Myocardial Ischemia and Infarction

Myocardial Infarction (MI) creates a hypoxic state, damaging cardiomyocytes and activating pro-inflammatory responses and fibroblasts, leading to fibrosis and adverse remodeling culminating in heart failure [77]. Across experimental models, EVs have emerged as key mediators of post-MI signaling through transfer of regulatory RNA cargo; however, the literature varies in EV source, injury model, and readouts, complicating direct comparison of efficacy and mechanism.

A consistent finding across multiple studies is EV-mediated cardiomyocyte–fibroblast crosstalk following ischemic injury. Cardiomyocyte-derived EVs enriched in long non-coding RNAs (lncRNAs) or microRNAs are internalized by cardiac fibroblasts, where they activate profibrotic gene programs while simultaneously influencing cardiomyocyte survival and post-infarction remodeling [78,79]. Although these studies converge on fibroblast reprogramming as a key EV-driven process, they differ in EV subtype, RNA class, and downstream functional emphasis, suggesting that distinct EV populations and cargo compositions may underlie similar pathological outcomes.

Beyond local myocardial signaling, EVs also contribute to systemic cardioprotection. Circulating EVs induced by remote ischemic conditioning (RIC) accumulate in damaged heart tissue post-MI and deliver regulatory microRNAs that engage Argonaute-2 dependent cardioprotective gene expression programs [80]. This mechanism contrasts with resident cardiac cell-derived EV signaling and highlights the contribution of systemic EV pools, whose cellular origin and targeting specificity remain incompletely defined.

EVs further play a significant regenerative role post-MI angiogenesis, proliferation, and cell survival. EVs derived from endothelial progenitor cells (EPCs), endothelial cells, cardiac progenitor cells, and pluripotent stem cell sources exert paracrine effects, essential for vascular regeneration, limiting fibrosis, and promoting cardiomyocyte proliferation through delivery of pro-regenerative RNA cargo [81,82,83,84]. While these studies report convergent functional outcomes, they differ substantially in EV cellular origin, cargo composition, and regenerative mechanisms, raising important considerations regarding scalability, safety, and translational relevance.

Additional investigations have linked EV cargo to regulation of angiogenesis, oxidative stress responses, autophagy, calcium homeostasis, and immune signaling, collectively contributing to myocardial protection and functional recovery after infarction [85,86,87,88,89,90]. Given the recurrent involvement of EV-associated microRNAs and other non-coding RNAs in these processes, key cargo, cellular resources, recipient cells, and functional outcomes are summarized in Table 3 to facilitate cross-study comparison and reduce redundancy.

In the context of ischemia/reperfusion (I/R) injury following revascularization, EV-based interventions have also demonstrated therapeutic benefit. Delivery of microRNA-enriched MSC-derived EVs improves cardiac function, reduces cardiac injury, promotes anti-inflammatory macrophage polarization, enhances angiogenesis, and reduces fibroblast proliferation, which collectively contribute to decreased CM apoptosis and inflammation [91]. These findings highlight context-dependent EV mechanisms distinct from MI-only models.

Despite strong preclinical evidence, several gaps remain. Variability in EV isolation, characterization, and dosing can alter cargo composition and biological activity, complicating cross-study comparisons. Moreover, many studies infer mechanisms from cargo enrichment without definitive causal validation, such as EV cargo depletion or pathway-specific inhibition in recipient cells. In addition, EV biodistribution, retention, and target engagement remain inconsistently assessed, particularly in large-animal models relevant to clinical translation. Addressing these limitations will be essential to advance EV-based therapies toward standardized and clinically translatable cardiac regeneration strategies.

**Table 3:** EV-associated non-coding RNAs involved in myocardial ischemia and infarction.

EV Source	RNA Cargo	Recipient Cell	Primary Function	Outcome Post-MI	Ref.
Cardiomyocytes	miR-195	Fibroblasts	Myofibroblast activation	Fibrosis regulation	[78]
Cardiomyocytes	miR-222, miR-143	Endothelial cells	Angiogenesis, proliferation	Cardioprotection	[85]
EPC (hypoxic)	miR-133	Endothelial cells	Angiogenesis	Vascular regeneration	[81,82]
Endothelial cells	circWhsc1	Cardiomyocytes	Proliferation	Cardiac regeneration	[83]
ESC-derived EVs	miR-294	CPCs, CMs	Survival, proliferation	Reduced fibrosis	[84]
Pericardial fluid EVs	let-7b-5p	Endothelial cells	TGFBR1 inhibition	Angiogenesis	[86]
ASCs	miR-31	Endothelial cells	HIF-1α activation	Ischemic angiogenesis	[87]
Hypoxic CMs	miR-30a	Cardiomyocytes	Autophagy regulation	Cell survival	[88]
EVs (post-MI)	miR-214	Cardiomyocytes	Ca^2+^ homeostasis	Reduced cell death	[89]
CPC-derived EVs	miR-201, miR-146a-3p, miR-132	Endothelial cells	Angiogenesis	Tissue repair	[90]
MSC-derived EVs	miR-125a-5p	Macrophages, CMs	Anti-inflammatory signaling	Reduced I/R injury	[91]
Cardiomyocyte EVs	lncRNAs (Neat1, ENSMUST00000122745)	Fibroblasts	Profibrotic signaling	Remodeling regulation	[79]
Circulating EVs (RIC)	miR-144	Cardiomyocytes	Gene regulation (Ago2)	Cardioprotection	[80]

Despite consistent cardioprotective outcomes across ischemic models, substantial variability in EV source, cargo composition, and experimental design limits cross-study comparability and hinders identification of conserved EV signatures required for clinical translation.

### 5.3. EVs in Cardiomyopathies

Cardiomyopathies are myocardial disorders that increase cardiac workload, impairing the heart’s ability to pump blood effectively [92]. Cardiomyopathies are classified into primary and secondary types. Primary cardiomyopathies can be idiopathic, genetic, acquired, or mixed, while secondary cardiomyopathies result from extrinsic factors such as hypertension, metabolic syndrome, CAD, ischemic heart disease, and drug-induced [93,94]. Growing evidence indicates that EVs contribute to cardiomyopathy pathogenesis in a disease subtype-specific manner, with differences in EV cellular origin, cargo composition, and functional impact.

#### 5.3.1. Hypertrophic Cardiomyopathy

Hypertrophic cardiomyopathy (HCM) is a common primary genetic disorder that follows an autosomal dominant pattern of inheritance, caused by mutations in sarcomeric genes resulting in unexplained left ventricular hypertrophy and diastolic dysfunction [95,96,97].

Given the growing interest in the role of EVs in HCM, several studies have investigated their molecular content and functional implications [98]. Transcriptomic analysis demonstrates that hypertrophic cardiomyocytes alter their EV content under increased workload, with enrichment of small non-coding nucleolar RNA (snoRNA) cargo and miRNAs, linked with dysregulated alternative splicing, metabolic stress, and hypertrophic signaling pathway [98]. In parallel, circulating EV profiling exhibits increased levels of platelet-derived EVs, progenitor endothelial cell-derived EVs, and neutrophil-derived EVs. While total EV concentration remains unchanged, these specific subpopulations were altered, correlating with impaired diastolic function and increased sudden cardiac death risk, supporting EVs as indicators of disease severity and progression rather than simple diagnostic markers [99].

Functionally, EV-mediated paracrine signaling in HCM converges on cardiomyocyte hypertrophy and fibroblast–cardiomyocyte crosstalk, predominantly driven by EV-associated miRNAs such as miR-21-3p and miR-27a, which target cytoskeletal and transcriptional regulators of hypertrophic growth [100,101]. Building on these insights, researchers have explored EV-based strategies as potential therapeutic interventions for HCM. Therapeutic proof-of-concept studies further demonstrate that engineered EV cargo, including the small RNA fragment YF1 delivered via cardiosphere-derived cell EVs, reduces hypertrophy, fibrosis, and inflammation while improving cardiac function in genetic HCM models [102].

#### 5.3.2. Dilated Cardiomyopathy

Dilated Cardiomyopathy (DCM) is characterized by the dilation of one or both ventricles along with impaired contractility, defined as a reduction in left ventricular ejection fraction (LVEF) to less than 40% [103]. DCM is commonly associated with enhanced susceptibility for arrhythmias, indicating that the pathogenesis of DCM involves the cardiac conduction system. As the disease progresses, both ventricles can fail, which can lead to heart failure (HF) and death [104,105].

EVs profiling based on sequencing of plasma-derived EVs from DCM patients exhibiting chronic heart failure (CHF) revealed extensive remodeling of EV-associated miRNA cargo compared to healthy controls, indicating a broad reprogramming of EV-mediated signaling in advanced disease states [106]. The results showed that 98 miRNAs were expressed with a significant difference between the two groups, with 50 miRNAs upregulated and 48 miRNAs downregulated. Six miRNAs have been identified to have major contributions to the development of DCM through different mechanisms, including collagen production and profibrotic activation of cardiac fibroblasts. These include miR-423-5p, hsa-miR-185-5p, hsa-miR-150-5p, and hsa-miR-10a-5p_R-1, which are involved in regulating fibrosis; hsa-miR-150-5p, which is associated with hypertrophy; hsa-miR-1304-3p_1ss13CA and hsa-miR-150-5p, which contribute to inflammation; hsa-miR-1304-3p_1ss13CA, which plays a role in oxidative stress; hsa-miR-150-5p, which affects angiogenesis; and sa-miR-3138_L-5R+2, which is linked to mitochondrial function [107].

To understand disease progression and clinical prognosis of patients with DCM, multiple studies have found reliable biomarkers and unique protein profiling. Proteomic analysis of plasma-derived EVs obtained from DCM observed a higher abundance of certain proteins in the cargo proteome of EVs compared to the control group. Among these, fibrinogen is linked to an increased risk of CVDs due to its role in endothelial injury, plasma viscosity, and thrombus formation [108,109,110]. Serotransferrin has also been associated with iron deficiency and overload and increased cardiovascular mortality [111,112,113]. In addition, elevated levels of protease inhibitor α-1-antitrypsin (AAT), a protein associated with heart failure, have been proposed as a biomarker reflecting disease status [114]. Consistent with these proteomic findings, serum exosomal miRNAs profiling revealed increased levels of exo-miR-92b-5p in DCM-AHF compared to the control group, suggesting its potential as a biomarker for predicting DCM-AHF [115].

Multiple studies have investigated the therapeutic potential of EVs in DCM. In experimental models of doxorubicin-induced DCM, intravenous administration of MSC-EVs improved cardiac function, attenuated cardiac dilation, reduced cardiomyocyte apoptosis, and decreased inflammatory cell infiltration [116]. Similarly, EVs derived from trophoblast stem cells mitigated cardiac dysfunction and inflammation through activation of the let-7i/YAP signaling pathway, highlighting a pro-survival and anti-inflammatory mechanism of action [117]. In addition, endothelial cell–derived EVs enriched in Krüppel-like factor 2-dependent cargo reduced cardiac inflammation and improved left ventricular function by targeting CCR2 and limiting Ly6C^high monocyte recruitment to the myocardium [118].

#### 5.3.3. Diabetic Cardiomyopathy

Diabetes mellitus (DM) affects approximately 9.3% of the global population [119]. In the United States, it affects about 38.4 million people and ranks as the eighth leading cause of death [120]. Diabetic cardiomyopathy (DmCM) is the myocardial structural and functional impairment as a result of diabetes and independent of other risk factors, such as coronary artery disease (CAD) or hypertension (HTN) [121,122]. DmCM is characterized by left ventricular dysfunction, myocardial fibrosis, and apoptosis, as well as coronary microvascular dysfunction, all increasing the risk of heart failure (HF) [123].

Early studies identified cardiomyocyte-derived exosomal miRNAs, particularly miR-1 and miR-133a, as being elevated in diabetic patients with myocardial steatosis and reduced cardiac output, supporting their potential utility as early biomarkers of metabolic myocardial stress [124]. In diabetic HFpEF, exosomal profiling further showed a characteristic miRNA shift, with miR-30d-5p and miR-126a-5p consistently reduced (both in small EVs and cardiac expression) and miR-34a-5p increased, while several additional exosomal miRNAs were also decreased [125]. Subsequent work linked miR-30d and miR-126a to loss of cardioprotective signaling and impaired vascular density, respectively, reinforcing their relevance to diabetic cardiac dysfunction [126,127,128]. However, partial overlap with non-diabetic heart failure signatures remains a limitation for disease specificity and supports the need for improved EV-based stratification in diabetic cardiomyopathy.

Beyond their role as biomarkers, EVs actively contribute to the pathogenesis of DmCM through maladaptive endothelial–cardiomyocyte and endothelial–fibroblast signaling. Under hyperglycemic conditions, cardiac microvascular endothelial cell-derived EVs are taken up by cardiomyocytes and fibroblasts, delivering pathogenic cargo such as Mst1 and TGF-β1 mRNA, which promotes cardiomyocyte apoptosis, suppresses autophagy, exacerbates insulin resistance, and drives interstitial and perivascular fibrosis [129,130]. These findings highlight endothelial-derived EVs as key mediators of metabolic and fibrotic remodeling in DmCM.

In contrast to these detrimental effects, researchers have also identified potential therapeutic strategies involving modifying EV cargo. Reduced levels of HSP20 in small EVs released from diabetic cardiomyocytes have been linked to disease progression, while restoration of HSP20 via engineered EVs attenuates cardiac dysfunction, hypertrophy, apoptosis, fibrosis, and microvascular rarefaction [131].

Similarly, MSC-derived EVs exert cardioprotective effects by inhibiting the TGF-β1/Smad2 pathway and improving fatty acid metabolism, thereby reducing myocardial fibrosis [132].

More recently, small EVs from ginsenoside RG1-stimulated MSCs were shown to deliver circNOTCH1 to macrophages, promoting M2 polarization and dampening maladaptive inflammatory responses in DmCM [133]. Collectively, these studies illustrate that while endogenous EV signaling under diabetic conditions contributes to myocardial injury, therapeutically engineered EVs can redirect these pathways toward repair, positioning EVs as both drivers of disease and promising targets for intervention in DmCM. Despite growing evidence for EV involvement in diabetic cardiomyopathy, the interplay between metabolic stress, endothelial-derived EV signaling, and cardiomyocyte dysfunction remains insufficiently characterized.

#### 5.3.4. Comparative Insights and Knowledge Gaps Across Cardiomyopathies

Taken together, EVs exhibit distinct pathogenic signatures across cardiomyopathy subtypes: HCM is dominated by hypertrophic and splicing-related RNA signaling, DCM by circulating EV biomarkers reflecting systemic inflammation and immune activation, and DmCM by metabolically driven endothelial-derived EV dysfunction. Despite overlapping downstream outcomes such as fibrosis and functional decline, the cellular origin, cargo drivers, and therapeutic leverage points differ substantially. Most studies remain largely descriptive, rely on heterogeneous EV isolation strategies, and lack rigorous causal validation of EV cargo, limiting cross-disease comparison and translational progress.

### 5.4. EVs in Atrial Fibrillation

Atrial fibrillation (AF) is the most common type of cardiac arrhythmia caused by abnormal electrical activity in the atria of the heart, causing them to fibrillate. The irregular rhythm in AF causes blood flow through the heart to become turbulent, increasing the chances of thrombus formation, which can dislodge, therefore making AF the leading cardiac cause of stroke [134,135]. Given its clinical significance, it is no surprise that numerous researchers have investigated the role of EVs in AF pathogenesis, progression, and therapeutic potential.

One of the major areas of investigation in AF is the miRNA cargo within EVs, which serves as a key molecular signature of disease progression. Exosomal miRNA profiling in AF patients compared with individuals in sinus rhythm has identified 39 dysregulated miRNAs, with three (miR-483-5p, miR-142-5p, miR-223-3p) being validated as potential biomarkers, particularly miR-483-5p, which showed a strong independent correlation with AF [136]. Additional studies elevated exosomal levels of miR-106b-3p, miR-590-5p, miR-339-3p, miR-378-3p, miR-328-3p, and miR-532-3p, linking these miRNAs to arrhythmogenesis, cell apoptosis, cell proliferation, oxygen homeostasis, and structural remodeling in AF [137]. Similarly, researchers identified significant differences in exosomal miRNA profiles between AF patients and sinus rhythm controls, with miR-124-3p, miR-378d, miR-2110, and miR-3180-3p being remarkably upregulated, while miR-223-5p, miR-574-3p, miR-125a-3p, and miR-1299 were downregulated [138]. These findings suggest that EVs carrying miRNA signatures play a crucial role in AF progression, influencing several key processes such as structural remodeling, apoptosis, and electrical signaling.

Beyond serving as biomarkers, certain miRNAs with exosomal cargo actively contribute to AF pathogenesis. For instance, miR-107 levels were found to be elevated in patients with AF, with USP14 identified as a direct target of this miRNA. Overexpression of miR-107 resulted in downregulation of USP14 and Bcl2, along with upregulation of ERK2, FAK, and Bax, suggesting its role in promoting cell viability, migration, apoptosis, and cell cycle progression [139]. In the context of postoperative AF (POAF), a common complication following cardiac surgery, elevated levels of miR-122-5p correlate with disease severity and prognosis, suggesting a role in perioperative inflammatory and stress responses [140]. Another key mechanism involves ferroptosis, an iron-dependent form of cell death. A study demonstrated that cardiac fibroblast-derived EVs promote ferroptosis in CMs by delivering miR-23-3p, which downregulates the protective gene SLC7A11 [141]. These findings highlight a novel mechanistic link between EVs and oxidative stress-induced cell death in AF.

Building on these mechanistic insights, EVs have also been explored as therapeutic targets and delivery platforms in AF. Direct administration of EVs into atrial tissue has been shown to reduce inflammation, atrial fibrosis, and hypertrophy caused by sterile pericarditis, suggesting the potential of EVs in the prevention of postoperative AF [142]. Modulation of EV cargo has further revealed therapeutic targets relevant to atrial remodeling. Atrial myocyte-derived EVs have increased levels of miR-210-3p compared to healthy controls, which promotes cell proliferation and collagen synthesis by inhibiting GPD1L in atrial fibroblasts, highlighting a potential therapeutic target for fibrosis-related AF progression [143]. Furthermore, myofibroblast-derived small EVs carry miR-21-3p, which downregulates the expression of the Cav1.2 calcium channel in CMs, a key factor in increasing AF susceptibility by altering ion channel expression [144]. EVs derived from epicardial adipose tissue harbored greater amounts of pro-inflammatory and profibrotic cytokines and miRNAs compared to a healthy control group, further promoting atrial fibrosis and inflammation [145].

Beyond EV delivery, targeting EV-associated signaling pathways has shown promise. Small EVs derived from Ang-II-treated human cardiac myocytes (Ang-II-Exo) contribute to AF progression by transferring the lncRNA PVT1 to macrophages, leading to M1 macrophage polarization by increasing IL-16 expression through the sponging of miR-145-5p, leading to extracellular matrix remodeling in atrial fibroblasts [146]. Pharmacological intervention can also modulate EV profiles. In patients with non-valvular AF, treatment with rivaroxaban, an anticoagulant with anti-inflammatory properties, compared to a control group on warfarin, was associated with a reduction in the expression of pro-inflammatory proteins in EVs and an increase in negative regulators of inflammation, highlighting the impact of the drug on the EV profile [147]. Similarly, inhibition of pro-fibrotic EV-associated pathways, including lncRNA NRON-miRNA-23 signaling, promotes M2 macrophage polarization and attenuates atrial fibrosis [148]. Engineered EV-based approaches have also been evaluated, including Nrf2-overexpressing BMSC-derived EVs, which were shown to reduce AF burden, cardiomyocyte apoptosis, and inflammation in experimental models [149]. These findings collectively highlight the growing potential of EVs as both therapeutic targets and treatment strategies in AF. However, most existing studies focus on EV-associated miRNAs as descriptive biomarkers or mechanistic mediators, whereas relatively few directly assess their predictive value or evaluate targeted therapeutic modulation in well-characterized patient cohorts.

### 5.5. EVs in Heart Failure

Heart failure (HF) is a life-threatening clinical syndrome caused by defects in the myocardium, leading to impaired ventricular filling or ejection. Affecting approximately 1–2% of the population, HF represents the end-stage of many cardiac diseases [150]. The main challenge with HF is its constant progression; therefore, early diagnosis is essential to prevent the worsening of the disease. Given this, EVs and their cargo have emerged as promising diagnostic and prognostic biomarkers for HF [151,152]. Several studies have reported significant differences in the quantity and composition of EVs in HF, originating from cardiomyocytes, immune cells, and endothelial cells. These findings suggest that EVs not only reflect the pathophysiological state of HF but may also contribute to disease progression and serve as potential therapeutic targets.

One of the major areas of EV research in HF focuses on exosomal miRNA expression profiles. Several miRNAs, including miR-194, miR-34a, and miR-192, are elevated in patients with heart failure following acute myocardial infarction (AMI) and are linked to p53-responsive stress pathways, suggesting a potential regulatory role in adverse remodeling [153]. In contrast, other EV-associated miRNAs, such as miR-27a, are reduced in HF patients but increase following treatment, suggesting diagnostic and prognostic value [154]. In like manner, miRNA-146a, which is induced in response to inflammation, was found to be elevated in HF patients, reinforcing the potential value of this and the above miRNAs as a biomarker for HF [155]. Large-scale profiling studies further demonstrate extensive remodeling of exosomal miRNA landscapes in HF, including in dilated cardiomyopathy-associated HF, emphasizing the robustness and the heterogeneity of EV-based RNA signatures.

Beyond diagnostic association, additional studies have focused on their functional implications in HF progression. Reductions in circulating miR-30d-5p and miR-126a-5p have been associated with impaired cardioprotective signaling and vascular dysfunction in HFpEF models [125]. Another study identified 32 significantly altered miRNAs in HF patients, with six (miR-210-3p, miR-22-5p, miR-22-3p, miR-21-3p, miR-339-3p, and miR-125a-5p) showing strong correlation with HF biomarkers, including NT-proBNP. Additionally, three miRNAs (miR-125a-5p, miR-10b-5p, and miR-9-5p) were linked to poor prognosis, as they were altered in patients expressing major clinical events [156]. However, many of these associations remain correlative, and overlap with other cardiac and systemic conditions highlights ongoing challenges in specificity.

In addition to RNA-based markers, protein cargo in circulating small EVs has provided insights into HF pathophysiology. Certain proteins, such as Cystatin C and CD14, have been associated with both HF and renal dysfunction, making them potential targets for disease prevention and treatment [157,158]. Immune cell-derived EVs are also increased in HF, particularly annexin-V+ (phosphatidylserine+)-positive EVs originating from neutrophils, monocytes, T-lymphocytes, and natural killer cells, with levels positively correlating with disease severity, as assessed by the New York Heart Association (NYHA) classification. These findings indicate that immune cell-derived EVs play an integral role in HF-associated inflammation.

Emerging evidence further implicates EVs in neurocardiac communication. Circulating EV-associated miRNAs can cross the blood-brain barrier (BBB) and impact central inflammation and sympathetic regulation. Specifically, exosomal miR-214-3p exacerbated inflammation in the rostral ventrolateral medulla (RVLM), while let-7g-5p and let-7i-5p exhibited protective anti-inflammatory effects [159]. In parallel, another study found that the cardiac-derived exosomal miRNAs from CHF models influence central inflammation via the Nrf2/antioxidant signaling pathway, further implicating EVs in modulating autonomic dysfunction in CHF [160].

Beyond their diagnostic and mechanistic roles, EVs are increasingly recognized for their therapeutic potential in HF treatment. Several miRNAs with antifibrotic roles, including miR-425 and miR-744, are decreased in HF, which is critical as these miRNAs normally have a protective role against fibrosis by suppressing TGFβ1 expression. Their reduction, therefore, contributes to the fibrogenesis seen in HF, suggesting their potential role as biomarkers for the disease [161]. Complementary evidence revealed that EVs derived from MSCs and cardiac progenitor cells exhibit immunomodulatory properties, inhibiting lymphocyte proliferation and antibody production, leading to a substantial reduction in IgG1, IgG4, and IgM levels, suggesting potential application for immune modulation in HF [162].

Further research into MSC-derived EVs (MSC-EVs) demonstrated their ability to reduce apoptosis and inflammation in cardiomyocytes with hypertrophy induced by angiotensin II (Ang II). MSC-EVs treatment resulted in downregulation of pro-apoptotic proteins Bax and caspase 3 and upregulation of the anti-apoptotic protein Bcl-2. It also reduced the levels of BNP and inflammatory cytokines IL-1β, IL-4, IL-6, and TNF-α. Moreover, it led to the downregulation of phosphorylated YAP (p-YAP) and upregulation of tafazzin (TAZ), indicating that MSC-EVs alleviate apoptosis and inflammation in cardiomyocytes by modulating the Hippo-YAP signaling pathway [163]. Similarly, EVs derived from bone marrow mesenchymal stem cells (BMSCs-EVs) in AMI-induced HF mouse models resulted in improved cardiac function, reduced myocardial fibrosis and inflammatory cell infiltration, increased angiogenesis, and inhibited apoptosis. The beneficial effects observed were mediated by the overexpression of bone morphogenic protein 2 (BMP2), as confirmed with reduced positive outcomes when BMP2 was inhibited [164].

Additionally, EVs derived from induced pluripotent stem cell-derived cardiovascular progenitors (iPSC-Pg) and cardiomyocytes (iPSC-CM) improved cardiac function and promoted tissue repair in mouse models with chronic heart failure (CHF). These EVs enhanced cell survival, angiogenesis, and proliferation while reducing fibrosis, inflammation, and apoptosis, largely attributed to their rich miRNA content associated with tissue repair pathways. Moreover, Zhong et al. found that HucMSC-EVs protected against doxorubicin (DOX)-induced heart failure by reducing oxidative stress, cell damage, and apoptosis. This protective effect was mediated through the miR-100-5p/NOX4 pathway, where miR-100-5p inhibited NOX4 expression [165]. Furthermore, a study investigated the effects of TSC-Exos treatment in doxorubicin-induced cardiotoxicity in mouse models. The results showed improved cardiac function and decreased cardiomyocyte apoptosis and mitochondrial fragmentation. The positive effects were attributed to improving mitochondrial fusion with increased Mfn2 expression [166]. Another study demonstrated that TSC-Exos mediates their therapeutic effect by increasing the expression of Zeb1 while inhibiting the expression of miR-200b, shedding light on the potential therapeutic effects of TSC-Exos in HF [167]. Collectively, these findings reinforce the growing role of EVs as both biomarkers and therapeutic agents in HF, highlighting their potential for future clinical applications in disease monitoring and treatment. However, because EV profiles in heart failure arise from multiple cardiac and systemic sources, standardized strategies to distinguish disease-specific EV signatures from secondary or comorbidity-driven signals remain limited. Representative EV sources, key molecular cargos, and their major functional and clinical implications across major cardiovascular diseases are summarized in Table 4.

**Table 4:** Representative extracellular vesicle sources, key cargos, and functional effects across major cardiovascular diseases.

CVD	EV Source	Key Cargo	Major Effect	Clinical Relevance	Ref.
MI	CM-EVs/MSC-EVs	miR-21, miR-210, miR-125a	Angiogenesis, survival	Biomarker therapy	[81,82,83,84,91]
HCM	Fibroblast-EVs	miR-21-3p, snoRNAs	Hypertrophy, fibrosis	Mechanistic	[98,99,100,101]
DCM	Plasma EVs	miR-423-5p, fibrinogen	Remodeling, inflammation	Biomarker	[108,109,111,112,113]
AF	Fibroblast-EVs	miR-21-3p, miR-23a	Electrical remodeling	Therapeutic target	[141,144]
HF	MSC-EVs	miR-425, miR-744	Anti-fibrotic	Therapy	[161,162,163,164]

Note: This table highlights representative EV-associated miRNAs and is not exhaustive. miRNAs were selected based on reproducibility and functional validation.

### 5.6. EVs-Mediated Angiogenesis in Cardiovascular Diseases

**Figure 4:**
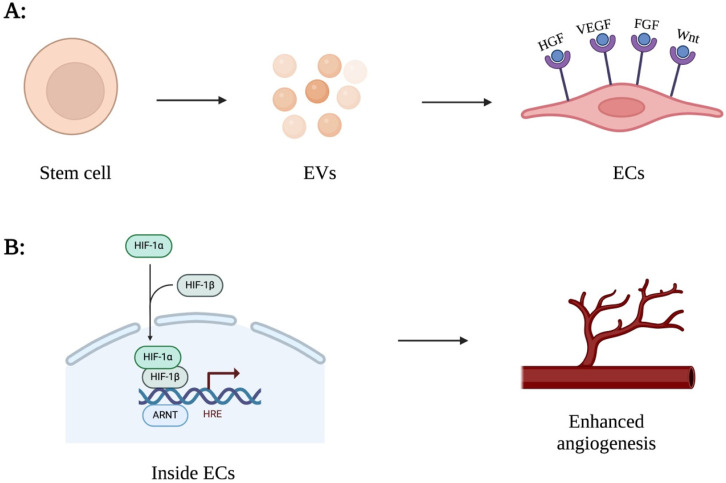
(**A**) Shows a stem cell releasing extracellular vesicles (EVs) that carry and release VEGF, HGF, FGF, and Wnt, which bind to receptors on ECs. (**B**) Illustrates the internal processes within ECs, highlighting the activation of the HIF pathway, resulting in enhanced angiogenesis.

Angiogenesis, the formation of new blood vessels from existing vasculature, is crucial in development, wound healing, and various physiological and pathological conditions. Recent studies have highlighted the significant role of EVs in modulating angiogenesis through their function as mediators of intercellular communication [168]. For instance, EV treatment has been shown to increase vascular endothelial growth factor (VEGF), hepatocyte growth factor (HGF), and fibroblast growth factor (FGF), all of which are vital mediators in the process of angiogenesis [169] (Figure 4).

Furthermore, research investigating the effects of EVs on cardiac function, blood circulation, and vessel formation in chronic myocardial ischemia has demonstrated multiple positive outcomes, including improvement of cardiac output and stroke volume, increased capillary and arteriolar density within the myocardium, and enhanced blood flow into ischemic tissues [170]. The pro-angiogenic effects of MSC-derived EVs have been linked to their release of signaling molecules that influence the local environment. Particularly, the increased expression of nuclear factor-kB (NF-kB) under hypoxic conditions and the transport of transcriptionally active STAT3 is associated with these effects [171,172]. These proteins augment the translation of pro-angiogenic proteins when transferred to endothelial cells.

Among the transferred proteins is Wnt, which promotes the transcription of molecules involved in angiogenesis by interacting with its receptor. EVs carry additional angiogenic-associated microRNAs that add to their therapeutic effect, such as miR-31 and miR-25. miR-31 acts by suppressing the factor-inhibiting HIF (FIH), while miR-25 promotes tip cell specification by reducing delta-like-4 (DLL4) [173]. Another miRNA, miR-125a, has also been found to promote angiogenesis by inhibiting DLL4 through its 3′ untranslated region, thereby promoting the formation of tip cells [174].

Further corroborating the importance of the Wnt pathway in EV angiogenic effects, one study demonstrated the role of human umbilical cord-mesenchymal stem cells-derived EVs in the promotion of the nuclear translocation of β-catenin in endothelial cells, leading to the activation of the Wnt/β-catenin signaling pathway, which is critical in the process of angiogenesis. Using a β-catenin inhibitor, ICG-001, reversed the activation of this pathway, confirming the importance of β-catenin and Wnt pathway in angiogenesis. Additionally, increased expression of pro-angiogenic proteins such as Proliferating Cell Nuclear Antigen (PCNA), Cyclin D3, N-cadherin, and β-catenin, alongside decreased expression of E-cadherin, which normally keeps the cells tightly bound, further supports the role of EVs in angiogenesis [175]. These findings emphasize the important role of EVs in angiogenesis and their potential use as therapeutic interventions in various cardiovascular diseases.

## 6. Advantages of EV Therapy over Cellular Therapy

Treating the heart with stem cells, particularly those of mesenchymal origin such as bone marrow-derived mesenchymal stem cells (BM-MSCs) and cardiac progenitor cells (CPCs), has been shown to increase LVEF, improve contractility, enhance angiogenesis, and reduce infarct size. BM-MSCs exhibited the ability to differentiate into cardiomyocytes, vascular smooth muscle cells, and endothelial cells in vitro models [176]. However, there is limited evidence supporting the capacity of these cells to differentiate in vivo. Additionally, MSCs typically have a limited survival time of around three weeks post-implantation, indicating that their therapeutic effects are mediated through intercellular mechanisms rather than direct differentiation.

The release of paracrine mediators such as cytokines, growth factors, and EVs by MSCs can be revolutionary in treating various CVDs. Among these, EVs have emerged as a promising therapeutic approach due to multiple advantages over direct cell transplantation [177,178,179,180]:

1. Reduced immunogenicity: EVs exhibit lower immunogenicity compared to cell-based treatments, due to their lower content of DNA and major histocompatibility complex (MHC) molecules.
2. Simplified Collection: The process of collecting EVs is less complex, less time-consuming, and more cost-effective compared to the isolation and preparation of MSCs.
3. Enhanced Storage Stability: EVs are more stable for long-term storage compared to MSCs. EVs can be stored at −20 °C for up to 1 week, and their biological activity is maintained during long-term storage at −80 °C.
4. Lack of Tumorigenicity: As EVs do not proliferate, this eliminates the risk of tumorigenicity, which is associated with MSC transplantation.
5. Effective Drug Delivery: EVs can be used as carriers for drugs and biological macromolecules, facilitating their transfer into recipient cells and enhancing intercellular communication.
6. Size Advantage: EVs are smaller than MSCs, allowing them to travel across capillaries without plugging them, which enhances their potential for systemic delivery.

## 7. Optimizing Therapeutic Potential of EVs

Given that the therapeutic value of EVs is cargo-dependent, modifying the EV content can significantly improve treatment outcomes. Current modification strategies can be divided into two categories: (1) modification of the EV content, which enhances its biological function and is executed indirectly at the level of the donor cell, and (2) modification of the EV surface, which enhances targeting specificity and is carried out at the level of the EV itself [181].

### 7.1. Indirect Modification of the EVs at the Level of the Donor Cells

One common way to optimize EV content is through a process called preconditioning. Preconditioning involves exposing donor cells to a stimulus during culturing to customize the cargo of the EVs. MSCs, for instance, can be primed with different stimuli, including cytokines or growth factors, hypoxia, pharmacological drugs, chemical agents, biomaterials, culture conditions, and other molecules [182].

#### 7.1.1. Cytokines or Growth Factors

Priming MSCs with cytokines or growth factors has been shown to increase the secretion of anti-inflammatory and immunomodulatory factors [183,184]. For example, bone marrow-derived mesenchymal stem cells (BM-MSCs) preconditioned with interferon-gamma (IFN-γ) and tumor necrosis factor-alpha (TNF-α) in vitro models suppressed T cell proliferation, induced greater differentiation of interleukin-10 (IL-10) secreting macrophages, and caused chromatin remodeling in the IDO1 promoter [185,186].

#### 7.1.2. Hypoxia

Hypoxia is another method of priming MSCs, and it has been shown to have several positive outcomes as it resembles their niche environment. Low oxygen levels have been associated with enhanced angiogenesis in vivo models through the modification of HIF-1α and Akt pathways, which promote the expression of pro-angiogenic and pro-survival genes. For example, in mouse models with radiation-induced lung injury, BM-MSCs exposed to hypoxic conditions showed increased survival, antioxidant ability, upregulated HIF-1α, and increased efficiency in treating radiation-induced lung injury [187].

#### 7.1.3. Pharmacological Agents

Pharmacological agents can be used to precondition MSCs, enhancing the therapeutic efficacy. For instance, preconditioning MSCs with 2,4-dinitrophenol (DNP) in infarcted rat models improved cardiac function, reduced scar formation, and increased expression of VEGF and HIF, as well as increased the expression of cardiomyogenic factors such as atrial natriuretic peptide (ANP), GATA-4, and Nkx2.5 [188]. Another study involving rat models with ischemic hearts treated with prolyl hydroxylase inhibition demonstrated enhanced angiogenic activities, reduced infarct size, decreased cell death, and upregulated survival [189].

#### 7.1.4. Biomaterials and Culture Conditions

Priming MSCs with biomaterials and various culture conditions has emerged as a promising approach to enhance therapeutic potential. Recent research has demonstrated that stimuli modulating three-dimensional (3D) culture significantly improved the functionality of MSCs derived from different sources. Several studies have reported improved angiogenic signaling, enhanced wound healing, increased cell survival, and optimized matrix production [190,191,192]. Additionally, spheroid formation using different techniques has yielded favorable outcomes, such as enhanced survival, proliferation, vascularization potential, improved healing, and increased expression of anti-inflammatory and anti-tumorigenic molecules compared to traditional two-dimensional (2D) models [193,194,195].

#### 7.1.5. Other Molecules

Priming MSCs with other molecules can maximize the therapeutic value mostly by enhancing the defense mechanisms against the detrimental effects of the host. For example, treating adipose tissue-derived MSCs in rat models with AMI with curcumin resulted in reduced fibrosis, increased viability, promoted neovascularization by upregulation of VEGF, and decreased myocardial apoptosis [196].

### 7.2. Direct EV Modification

**Figure 5:**
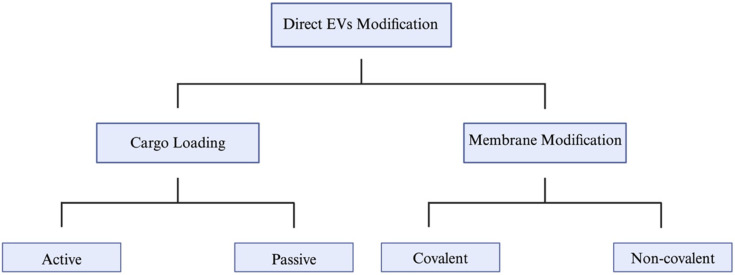
Diagram illustrating the direct modification methods of extracellular vesicles (EVs), categorized into cargo loading (active and passive) and membrane modification (covalent and non-covalent). Created with Biorender.com.

Direct modification of EVs is simpler, more rapid, and more versatile compared to engineering the parent cells [197]. This approach provides a straightforward pathway for the introduction of desired features. Certain active modification methods can help avoid the incompetent incorporations seen in certain cell-based methods, ensuring that the modifications are effective and functional [198]. Direct modification can be carried out by two primary approaches: cargo loading or direct membrane modifications (Figure 5).

#### 7.2.1. Cargo Loading

The incorporation of the therapeutic agent into the EV can be done either passively or actively. Passive modification mainly relies on the incorporation of the cargo without the need for an external force. Generally, hydrophobic molecules interact with the lipid bilayer of the EVs’ membrane, making passive co-incubation the best approach for hydrophobic drugs with low solubility, thus providing longevity to the half-life of the drug [199]. Another passive modification approach involves using electrostatic forces, as EVs have a negatively charged membrane, utilizing cationic lipids or pH-fusogenic peptides can improve the release of EV contents into the cytosol [200,201].

Active loading methods refer to methods in which the therapeutic agent is introduced across the EV membrane by electroporation, sonication, freeze-thaw cycles, extrusion, and similar techniques [202,203,204,205]. Research comparing these methods suggests that all active loading methods are more efficient than passive ones, particularly sonication and extrusion [206]. However, these methods have some limitations; for example, electroporation and freeze-thaw cycles can cause EV aggregation in the media containing phosphate-buffered saline or sucrose [207,208]. Additionally, extrusion methods can damage EV membrane properties. Aggregation has also been observed in freeze-thaw cycles and sonication methods. Based on the research presented, the most appropriate loading method for a certain drug depends on its properties. For example, electroporation is suitable for small RNA cargos due to its higher loading efficiency, while extrusion and sonication are more appropriate for larger proteins and hydrophilic molecules [209,210]. Passive loading methods are suitable for small molecules and hydrophobic drugs that can cross the hydrophobic EV membranes.

#### 7.2.2. Modification of EV Membrane

Direct modification of the EV membrane can be divided into two main categories: covalent and non-covalent modifications. Covalent modifications enable functional groups to form covalent bonds with the EVs. For instance, sulfhydryl groups are abundantly present on the surface of EVs, making them suitable binding sites for EV labeling. This can be achieved through the Michael addition reaction between maleimide and sulfhydryl.

Non-covalent modifications depend on the characteristics of the EVs. As the EV membranes primarily consist of amphiphilic structures such as phospholipids, cholesterol, and glycolipids, hydrophobic compounds can integrate into the EV membranes through hydrophobic interactions. Additionally, the negatively charged surface of EVs allows positively charged molecules to bind via electrostatic interactions [211]. Moreover, ligand-coupled molecules can bind to their respective receptors present on the surface of EVs [212].

## 8. Challenges in the Use of EVs

There are significant challenges in the clinical application of EVs for therapeutic purposes, and research is ongoing to confirm their readiness for patient use [213]. The National Heart, Lung, and Blood Institute (NHLBI) held a workshop where the pros and cons of EVs were discussed. Dr. Philip Yang highlighted important obstacles, including the EV population and cargo heterogeneity, and limited knowledge in pharmacological applications such as dosing, delivery route, biodistribution, pharmacokinetics, and pharmacodynamics. Additionally, the lack of standardized engineering methods for isolation and drug loading poses a significant challenge [214].

Another important challenge is the storage of EVs, as it can lead to temperature-induced damage from freezing and thawing and potential leakage of their contents over time. Studies have shown that storage conditions can alter the size and morphology of EVs, as well as their RNA and protein content. For instance, small EVs derived from mouse bronchoalveolar lavage fluid exhibited a 10% increase in diameter and reduced charge density when stored at +4 °C, while those stored at −80 °C showed a 25% increase in diameter and a more significant reduction in charge density [215].

Several factors, such as passage number, seeding densities, glucose conditions, and antibiotic use in the culture medium, can contaminate or alter the cargo content of EVs. The lack of an EV dosage guide for patients, derived from pre-clinical models’ biodistribution patterns, adds to the complexity. Additionally, potential risks in EV therapy include the possibility of thrombosis, hemostatic disturbances, alloimmune responses, and the elimination of EVs by the reticuloendothelial system [216].

An important obstacle in the clinical use of EVs is the route of administration. For example, a study conducted in 2022 visualized MSC-EV following intramyocardial and intravenous injections in mouse models with infarcted hearts, aiming to compare the myocardial uptake following both routes. The results showed effective uptake of EVs following intramyocardial injection but no uptake following intravenous injection. This poses a significant challenge, as many patients with heart diseases also suffer from heart failure, making them fragile and potentially unable to tolerate the invasiveness of thoracotomy required for intramyocardial injection.

Despite the growing therapeutic interest in extracellular vesicles, several challenges continue to limit their clinical translation, including scalable and reproducible manufacturing, standardized dosing strategies, and an incomplete understanding of EV biodistribution and persistence in vivo. Variability in EV cargo arising from differences in cell source, isolation methods, and culture conditions further complicates cross-study comparison and therapeutic consistency. In addition, long-term safety, immunogenicity, off-target effects, and regulatory requirements for product characterization and batch-to-batch consistency remain critical barriers to routine clinical application.

## 9. Conclusions and Future Perspective

Extracellular vesicles (EVs) have emerged as important mediators in various cardiovascular diseases (CVDs), with substantial evidence supporting their potential use as biomarkers for disease prediction and severity assessment, as well as therapeutic agents. Despite the advancement in knowledge of EVs in CVDs, clinical translation remains limited, with a persistent gap between promising preclinical findings and validated patient-based applications. While preclinical studies have shown encouraging results, the clinical application of EVs remains underexplored, underscoring the need for further studies to translate preclinical success into clinical practice.

Future research should focus on large-scale, well-designed clinical trials to validate EV-based biomarkers for early diagnosis and prognosis, ensuring their reproducibility and clinical utility, with careful consideration of patient stratification and clinically meaningful endpoints. Additionally, optimizing EV isolation and characterization techniques is critical to achieving standardized protocols for clinical use.

Robust in vivo tracking strategies will be necessary to better define EV biodistribution, tissue targeting, persistence, and clearance following administration, while investigating the long-term safety, biodistribution, and potential off-target effects of EV-based therapies will be essential for their regulatory approval. Translational studies in large-animal models that more closely reflect human cardiovascular physiology may further help bridge the gap between preclinical findings and clinical application. Moreover, integrating EVs into precision medicine approaches, including patient-specific therapies and targeted drug delivery systems, could significantly enhance treatment efficacy in CVDs by improving tissue specificity and therapeutic targeting. Collaborative efforts between researchers, clinicians, and industry partners will be key to accelerating the translation of EV research into real-world applications.

## Data Availability

No new data were created or analyzed in this study.

## References

[1] Yuan H.L., Chang L., Fan W.W., Liu X., Li Q., Tian C., Zhao J., Li Z.-A., Pan X.-H., Zhu X.-Q. (2024). Application and challenges of stem cells in cardiovascular aging. Regen. Ther..

[2] Karantalis V., Hare J.M. (2015). Use of mesenchymal stem cells for therapy of cardiac disease. Circ. Res..

[3] Quinn C., Flake A.W. (2008). In vivo Differentiation Potential of Mesenchymal Stem Cells: Prenatal and Postnatal Model Systems. Transfus. Med. Hemother..

[4] Phinney D.G., Prockop D.J. (2007). Concise Review: Mesenchymal Stem/Multipotent Stromal Cells: The State of Transdifferentiation and Modes of Tissue Repair—Current Views. Stem Cells.

[5] D’souza N., Burns J.S., Grisendi G., Candini O., Veronesi E., Piccinno S., Horwitz E.M., Paolucci P., Conte P., Dominici M. (2013). MSC and Tumors: Homing, Differentiation, and Secretion Influence Therapeutic Potential. Adv. Biochem. Eng. Biotechnol..

[6] Biehl J.K., Russell B. (2009). Introduction to stem cell therapy. J. Cardiovasc. Nurs..

[7] Kahrizi M.S., Mousavi E., Khosravi A., Rahnama S., Salehi A., Nasrabadi N., Ebrahimzadeh F., Jamali S. (2023). Recent advances in pre-conditioned mesenchymal stem/stromal cell (MSCs) therapy in organ failure; a comprehensive review of preclinical studies. Stem Cell Res. Ther..

[8] Patel A.N., Bartlett C.E., Ichim T.E., Perin E.C., Miller L.W., Taylor D.A., Willerson J.T. (2016). Mesenchymal stem cells. Stem Cell and Gene Therapy for Cardiovascular Disease.

[9] Barry F.P., Murphy J.M. (2004). Mesenchymal stem cells: Clinical applications and biological characterization. Int. J. Biochem. Cell Biol..

[10] Cona L. (2024). Types of Mesenchymal Stem Cells (MSCs).

[11] Rani S., Ryan A.E., Griffin M.D., Ritter T. (2015). Mesenchymal Stem Cell-derived Extracellular Vesicles: Toward Cell-free Therapeutic Applications. Mol. Ther..

[12] Liau L.L., Looi Q.H., Chia W.C., Subramaniam T., Ng M.H., Law J.X. (2020). Treatment of spinal cord injury with mesenchymal stem cells. Cell Biosci..

[13] Yáñez-Mó M., Siljander P.R.-M., Andreu Z., Bedina Zavec A., Borràs F.E., Buzas E.I., Buzas K., Casal E., Cappello F., Carvalho J. (2015). Biological properties of extracellular vesicles and their physiological functions. J. Extracell. Vesicles.

[14] Hade M.D., Suire C.N., Suo Z. (2021). Mesenchymal Stem Cell-Derived Exosomes: Applications in Regenerative Medicine. Cells.

[15] Zhou X., Liu S., Lu Y., Wan M., Cheng J., Liu J. (2023). MitoEVs: A new player in multiple disease pathology and treatment. J. Extracell. Vesicles.

[16] Maumus M., Rozier P., Boulestreau J., Jorgensen C., Noël D. (2020). Mesenchymal Stem Cell-Derived Extracellular Vesicles: Opportunities and Challenges for Clinical Translation. Front. Bioeng. Biotechnol..

[17] Doyle L., Wang M. (2019). Overview of Extracellular Vesicles, Their Origin, Composition, Purpose, and Methods for Exosome Isolation and Analysis. Cells.

[18] Rautou P.E., Vion A.C., Amabile N., Chironi G., Simon A., Tedgui A., Boulanger C.M. (2011). Microparticles, Vascular Function, and Atherothrombosis. Circ. Res..

[19] Kowal J., Arras G., Colombo M., Jouve M., Morath J.P., Primdal-Bengtson B., Dingli F., Loew D., Tkach M., Théry C. (2016). Proteomic comparison defines novel markers to characterize heterogeneous populations of extracellular vesicle subtypes. Proc. Natl. Acad. Sci. USA.

[20] Xu C.M., Sabe S.A., Brinck-Teixeira R., Sabra M., Sellke F.W., Abid M.R. (2023). Visualization of cardiac uptake of bone marrow mesenchymal stem cell-derived extracellular vesicles after intramyocardial or intravenous injection in murine myocardial infarction. Physiol. Rep..

[21] Xu C.M., Karbasiafshar C., Brinck-Teixeira R., Broadwin M., Sellke F.W., Abid M.R. (2023). Diabetic state of human coronary artery endothelial cells results in altered effects of bone mesenchymal stem cell-derived extracellular vesicles. Physiol. Rep..

[22] Jeppesen D.K., Fenix A.M., Franklin J.L., Higginbotham J.N., Zhang Q., Zimmerman L.J., Liebler D.C., Ping J., Liu Q., Evans R. (2019). Reassessment of Exosome Composition. Cell.

[23] Karbasiafshar C., Sellke F.W., Abid M.R. (2021). Mesenchymal stem cell-derived extracellular vesicles in the failing heart: Past, present, and future. Am. J. Physiol. Heart Circ. Physiol..

[24] Lötvall J., Hill A.F., Hochberg F., Buzás E.I., Di Vizio D., Gardiner C., Gho Y.S., Kurochkin I.V., Mathivanan S., Quesenberry P. (2014). Minimal experimental requirements for definition of extracellular vesicles and their functions: A position statement from the International Society for Extracellular Vesicles. J. Extracell. Vesicles.

[25] Sheta M., Taha E.A., Lu Y., Eguchi T. (2023). Extracellular Vesicles: New Classification and Tumor Immunosuppression. Biology.

[26] Hornick N.I., Huan J., Doron B., Goloviznina N.A., Lapidus J., Chang B.H., Kurre P. (2015). Serum Exosome MicroRNA as a Minimally-Invasive Early Biomarker of AML. Sci. Rep..

[27] Akers J.C., Ramakrishnan V., Kim R., Skog J., Nakano I., Pingle S., Kalinina J., Hua W., Kesari S., Mao Y. (2013). MiR-21 in the extracellular vesicles (EVs) of cerebrospinal fluid (CSF): A platform for glioblastoma biomarker development. PLoS ONE.

[28] Shi R., Wang P.Y., Li X.Y., Chen J.X., Li Y., Zhang X.Z., Zhang C.G., Jiang T., Li W.B., Ding W. (2015). Exosomal levels of miRNA-21 from cerebrospinal fluids associated with poor prognosis and tumor recurrence of glioma patients. Oncotarget.

[29] Pisitkun T., Shen R.F., Knepper M.A. (2004). Identification and proteomic profiling of exosomes in human urine. Proc. Natl. Acad. Sci. USA.

[30] Vojtech L., Woo S., Hughes S., Levy C., Ballweber L., Sauteraud R.P., Strobl J., Westerberg K., Gottardo R., Tewari M. (2014). Exosomes in human semen carry a distinctive repertoire of small non-coding RNAs with potential regulatory functions. Nucleic Acids Res..

[31] Zlotogorski-Hurvitz A., Dayan D., Chaushu G., Korvala J., Salo T., Sormunen R., Vered M. (2015). Human saliva-derived exosomes: Comparing methods of isolation. J. Histochem. Cytochem..

[32] Dixon C.L., Sheller-Miller S., Saade G.R., Fortunato S.J., Lai A., Palma C., Guanzon D., Salomon C., Menon R. (2018). Amniotic Fluid Exosome Proteomic Profile Exhibits Unique Pathways of Term and Preterm Labor. Endocrinology.

[33] Milasan A., Tessandier N., Tan S., Brisson A., Boilard E., Martel C. (2016). Extracellular vesicles are present in mouse lymph and their level differs in atherosclerosis. J. Extracell. Vesicles.

[34] Yoon S.B., Chang J.H. (2017). Extracellular vesicles in bile: A game changer in the diagnosis of indeterminate biliary stenoses?. Hepatobiliary Surg. Nutr..

[35] Colombo M., Moita C., van Niel G., Kowal J., Vigneron J., Benaroch P., Manel N., Moita L.F., Théry C., Raposo G. (2013). Analysis of ESCRT functions in exosome biogenesis, composition and secretion highlights the heterogeneity of extracellular vesicles. J. Cell Sci..

[36] Trajkovic K., Hsu C., Chiantia S., Rajendran L., Wenzel D., Wieland F., Simons M. (2008). Ceramide Triggers Budding of Exosome Vesicles into Multivesicular Endosomes. Science.

[37] Simons M., Raposo G. (2009). Exosomes—Vesicular carriers for intercellular communication. Curr. Opin. Cell Biol..

[38] Borges F.T., Melo S.A., Özdemir B.C., Kato N., Revuelta I., Miller C.A., Gattone V.H., LeBleu V.S., Kalluri R. (2013). TGF-β1-containing exosomes from injured epithelial cells activate fibroblasts to initiate tissue regenerative responses and fibrosis. J. Am. Soc. Nephrol..

[39] Raposo G., Stoorvogel W. (2013). Extracellular vesicles: Exosomes, microvesicles, and friends. J. Cell Biol..

[40] Babst M., Katzmann D.J., Estepa-Sabal E.J., Meerloo T., Emr S.D. (2002). ESCRT-III: An endosome-associated heterooligomeric protein complex required for MVB sorting. Dev. Cell..

[41] Morita E., Sandrin V., Chung H.Y., Morham S.G., Gygi S.P., Rodesch C.K., Sundquist W.I. (2007). Human ESCRT and ALIX proteins interact with proteins of the midbody and function in cytokinesis. EMBO J..

[42] Stuffers S., Sem Wegner C., Stenmark H., Brech A. (2009). Multivesicular Endosome Biogenesis in the Absence of ESCRTs. Traffic.

[43] Theos A.C., Truschel S.T., Tenza D., Hurbain I., Harper D.C., Berson J.F., Thomas P.C., Raposo G., Marks M.S. (2006). A lumenal domain-dependent pathway for sorting to intralumenal vesicles of multivesicular endosomes involved in organelle morphogenesis. Dev. Cell.

[44] van Niel G., Charrin S., Simoes S., Romao M., Rochin L., Saftig P., Marks M.S., Rubinstein E., Raposo G. (2011). The tetraspanin CD63 regulates ESCRT-independent and -dependent endosomal sorting during melanogenesis. Dev. Cell.

[45] Escola J.M., Kleijmeer M.J., Stoorvogel W., Griffith J.M., Yoshie O., Geuze H.J. (1998). Selective Enrichment of Tetraspan Proteins on the Internal Vesicles of Multivesicular Endosomes and on Exosomes Secreted by Human B-lymphocytes. J. Biol. Chem..

[46] Möbius W., Ohno-Iwashita Y., van Donselaar E.G., Oorschot V.M.J., Shimada Y., Fujimoto T., Heijnen H.F.G., Geuze H.J., Slot J.W. (2002). Immunoelectron Microscopic Localization of Cholesterol Using Biotinylated and Non-cytolytic Perfringolysin O. J. Histochem. Cytochem..

[47] Heijnen H.F., Schiel A.E., Fijnheer R., Geuze H.J., Sixma J.J. (1999). Activated platelets release two types of membrane vesicles: Microvesicles by surface shedding and exosomes derived from exocytosis of multivesicular bodies and alpha-granules. Blood.

[48] Correll V.L., Otto J.J., Risi C.M., Main B.P., Boutros P.C., Kislinger T., Galkin V.E., Nyaldhiwe J.O., Semmes O.J., Yang L. (2022). Optimization of small extracellular vesicle isolation from expressed prostatic secretions in urine for in-depth proteomic analysis. J. Extracell. Vesicles.

[49] Christianson H.C., Svensson K.J., van Kuppevelt T.H., Li J.P., Belting M. (2013). Cancer cell exosomes depend on cell-surface heparan sulfate proteoglycans for their internalization and functional activity. Proc. Natl. Acad. Sci. USA.

[50] Wickman G., Julian L., Olson M.F. (2012). How apoptotic cells aid in the removal of their own cold dead bodies. Cell Death Differ..

[51] Théry C., Boussac M., Véron P., Ricciardi-Castagnoli P., Raposo G., Garin J., Amigorena S. (2001). Proteomic Analysis of Dendritic Cell-Derived Exosomes: A Secreted Subcellular Compartment Distinct from Apoptotic Vesicles. J. Immunol..

[52] Zhang H., Freitas D., Kim H.S., Fabijanic K., Li Z., Chen H., Mark M.T., Molina H., Martin A.B., Bojmar L. (2018). Identification of distinct nanoparticles and subsets of extracellular vesicles by asymmetric flow field-flow fractionation. Nat. Cell Biol..

[53] Böing A.N., van der Pol E., Grootemaat A.E., Coumans F.A.W., Sturk A., Nieuwland R. (2014). Single-step isolation of extracellular vesicles by size-exclusion chromatography. J. Extracell. Vesicles.

[54] Cheruvanky A., Zhou H., Pisitkun T., Kopp J.B., Knepper M.A., Yuen P.S.T., Star R.A. (2007). Rapid isolation of urinary exosomal biomarkers using a nanomembrane ultrafiltration concentrator. Am. J. Physiol.-Ren. Physiol..

[55] Clos-Sansalvador M., Monguió-Tortajada M., Roura S., Franquesa M., Borràs F.E. (2022). Commonly used methods for extracellular vesicles’ enrichment: Implications in downstream analyses and use. Eur. J. Cell Biol..

[56] Zhang Q., Higginbotham J.N., Jeppesen D.K., Yang Y.P., Li W., McKinley E.T., Graves-Deal R., Ping J., Britain C.M., Dorsett K.A. (2019). Transfer of Functional Cargo in Exomeres. Cell Rep..

[57] Zhang Q., Jeppesen D.K., Higginbotham J.N., Graves-Deal R., Trinh V.Q., Ramirez M.A., Sohn Y., Neininger A.C., Taneja N., McKinley E.T. (2021). Supermeres are functional extracellular nanoparticles replete with disease biomarkers and therapeutic targets. Nat. Cell Biol..

[58] Konoshenko M.Y., Lekchnov E.A., Vlassov A.V., Laktionov P.P. (2018). Isolation of Extracellular Vesicles: General Methodologies and Latest Trends. Biomed. Res. Int..

[59] Yamamoto T., Kosaka N., Ochiya T. (2019). Latest advances in extracellular vesicles: From bench to bedside. Sci. Technol. Adv. Mater..

[60] Coulter B. Isolation Methods for Extracellular Vesicles. https://www.beckman.com/resources/sample-type/extracellular-vesicles/getting-started/isolation/isolation-methods.

[61] Monguió-Tortajada M., Gálvez-Montón C., Bayes-Genis A., Roura S., Borràs F.E. (2019). Extracellular vesicle isolation methods: Rising impact of size-exclusion chromatography. Cell. Mol. Life Sci..

[62] Williams S., Fernandez-Rhodes M., Law A., Peacock B., Lewis M.P., Davies O.G. (2023). Comparison of extracellular vesicle isolation processes for therapeutic applications. J. Tissue Eng..

[63] Mercadal M., Herrero C., López-Rodrigo O., Castells M., de la Fuente A., Vigués F., Oliva R. (2020). Impact of Extracellular Vesicle Isolation Methods on Downstream miRNA Analysis in Semen: A Comparative Study. Int. J. Mol. Sci..

[64] Benedikter B.J., Bouwman F.G., Vajen T., Heinzmann A.C.A., Grauls G., Mariman E.C., Wouters E.F.M., Savelkoul P.H., Lopez-Iglesias C., Koenen R.R. (2017). Ultrafiltration combined with size exclusion chromatography efficiently isolates extracellular vesicles from cell culture media for compositional and functional studies. Sci. Rep..

[65] Fu S., Zhang Y., Li Y., Luo L., Zhao Y., Yao Y. (2020). Extracellular vesicles in cardiovascular diseases. Cell Death Discov..

[66] Pang J.L., Shao H., Xu X.G., Lin Z.W., Chen X.Y., Chen J.Y., Mou X.-Z., Hu P.-Y. (2024). Targeted drug delivery of engineered mesenchymal stem/stromal-cell-derived exosomes in cardiovascular disease: Recent trends and future perspectives. Front. Bioeng. Biotechnol..

[67] Boulanger C.M., Loyer X., Rautou P.E., Amabile N. (2017). Extracellular vesicles in coronary artery disease. Nat. Rev. Cardiol..

[68] Chen M., Wu Y., Chen C. (2025). Extracellular Vesicles as Emerging Regulators in Ischemic and Hypertrophic Cardiovascular Diseases: A Review of Pathogenesis and Therapeutics. Med Sci. Monit..

[69] Wang J., Tan G.J., Han L.N., Bai Y.Y., He M., Liu H.B. (2017). Novel biomarkers for cardiovascular risk prediction. J. Geriatr. Cardiol..

[70] Dhingra R., Vasan R.S. (2017). Biomarkers in cardiovascular disease: Statistical assessment and section on key novel heart failure biomarkers. Trends Cardiovasc. Med..

[71] Dickhout A., Koenen R.R. (2018). Extracellular Vesicles as Biomarkers in Cardiovascular Disease; Chances and Risks. Front. Cardiovasc. Med..

[72] Laura Francés J., Pagiatakis C., Di Mauro V., Climent M. (2023). Therapeutic Potential of EVs: Targeting Cardiovascular Diseases. Biomedicines.

[73] Puhm F., Boilard E., Machlus K.R. (2021). Platelet Extracellular Vesicles: Beyond the Blood. Arterioscler. Thromb. Vasc. Biol..

[74] Niu C., Wang X., Zhao M., Cai T., Liu P., Li J., Willard B., Zu L., Zhou E., Li Y. (2016). Macrophage Foam Cell-Derived Extracellular Vesicles Promote Vascular Smooth Muscle Cell Migration and Adhesion. J. Am. Heart Assoc..

[75] Nozaki T., Sugiyama S., Sugamura K., Ohba K., Matsuzawa Y., Konishi M., Matsubara J., Akiyama E., Sumida H., Matsui K. (2010). Prognostic value of endothelial microparticles in patients with heart failure. Eur. J. Heart Fail..

[76] de Freitas R.C.C., Hirata R.D.C., Hirata M.H., Aikawa E. (2021). Circulating Extracellular Vesicles As Biomarkers and Drug Delivery Vehicles in Cardiovascular Diseases. Biomolecules.

[77] Bheri S., Kassouf B.P., Park H.J., Hoffman J.R., Davis M.E. (2021). Engineering Cardiac Small Extracellular Vesicle-Derived Vehicles with Thin-Film Hydration for Customized microRNA Loading. J. Cardiovasc. Dev. Dis..

[78] Morelli M.B., Shu J., Sardu C., Matarese A., Santulli G. (2019). Cardiosomal microRNAs Are Essential in Post-Infarction Myofibroblast Phenoconversion. Int. J. Mol. Sci..

[79] Kenneweg F., Bang C., Xiao K., Boulanger C.M., Loyer X., Mazlan S., Schroen B., Hermans-Beijnsberger S., Foinquinos A., Hirt M.N. (2019). Long Noncoding RNA-Enriched Vesicles Secreted by Hypoxic Cardiomyocytes Drive Cardiac Fibrosis. Mol. Ther. Nucleic Acids.

[80] Lassen T.R., Just J., Hjortbak M.V., Jespersen N.R., Stenz K.T., Gu T., Yan Y., Su J., Hansen J., Bæk R. (2021). Cardioprotection by remote ischemic conditioning is transferable by plasma and mediated by extracellular vesicles. Basic. Res. Cardiol..

[81] Terriaca S., Fiorelli E., Scioli M.G., Fabbri G., Storti G., Cervelli V., Orlandi A. (2021). Endothelial Progenitor Cell-Derived Extracellular Vesicles: Potential Therapeutic Application in Tissue Repair and Regeneration. Int. J. Mol. Sci..

[82] Lin F., Zeng Z., Song Y., Li L., Wu Z., Zhang X., Li Z., Ke X., Hu X. (2019). YBX-1 mediated sorting of miR-133 into hypoxia/reoxygenation-induced EPC-derived exosomes to increase fibroblast angiogenesis and MEndoT. Stem Cell Res. Ther..

[83] Wei G., Li C., Jia X., Xie J., Tang Z., Jin M., Chen Q., Sun Y., He S., Li X. (2023). Extracellular vesicle-derived CircWhsc1 promotes cardiomyocyte proliferation and heart repair by activating TRIM59/STAT3/Cyclin B2 pathway. J. Adv. Res..

[84] Khan M., Nickoloff E., Abramova T., Johnson J., Verma S.K., Krishnamurthy P., Mackie S., Vaughan M., Garikipati R.L., Benedict C. (2015). Embryonic Stem Cell–Derived Exosomes Promote Endogenous Repair Mechanisms and Enhance Cardiac Function Following Myocardial Infarction. Circ. Res..

[85] Ribeiro-Rodrigues T.M., Laundos T.L., Pereira-Carvalho R., Batista-Almeida D., Pereira R., Coelho-Santos V., Silva A.P., Fernandes R., Zuzarte M., Enguita F.J. (2017). Exosomes secreted by cardiomyocytes subjected to ischaemia promote cardiac angiogenesis. Cardiovasc. Res..

[86] Alam P., Maliken B.D., Jones S.M., Ivey M.J., Wu Z., Wang Y., Kanisicak O. (2021). Cardiac Remodeling and Repair: Recent Approaches, Advancements, and Future Perspective. Int. J. Mol. Sci..

[87] Zhu D., Wang Y., Thomas M., McLaughlin K., Oguljahan B., Henderson J., Yang Q., Chen Y.E., Liu D. (2022). Exosomes from adipose-derived stem cells alleviate myocardial infarction via microRNA-31/FIH1/HIF-1α pathway. J. Mol. Cell. Cardiol..

[88] Rezaie J., Rahbarghazi R., Pezeshki M., Mazhar M., Yekani F., Khaksar M., Shokrallahi E., Amini H., Hashemzadeh S., Sokullu S.E. (2019). Cardioprotective role of extracellular vesicles: A highlight on exosome beneficial effects in cardiovascular diseases. J. Cell. Physiol..

[89] Jadli A.S., Parasor A., Gomes K.P., Shandilya R., Patel V.B. (2021). Exosomes in Cardiovascular Diseases: Pathological Potential of Nano-Messenger. Front. Cardiovasc. Med..

[90] Davidson S.M., Andreadou I., Barile L., Birnbaum Y., Cabrera-Fuentes H.A., Cohen M.V., Downey J.M., Girão H., Pagliaro P., Penna C. (2019). Circulating blood cells and extracellular vesicles in acute cardioprotection. Cardiovasc. Res..

[91] Gao L., Qiu F., Cao H., Li H., Dai G., Ma T., Gong Y., Luo W., Zhu D., Qiu Z. (2023). Therapeutic delivery of microRNA-125a-5p oligonucleotides improves recovery from myocardial ischemia/reperfusion injury in mice and swine. Theranostics.

[92] Mayo Clinic Cardiomyopathy: Symptoms and Causes. https://www.mayoclinic.org/diseases-conditions/cardiomyopathy/symptoms-causes/syc-20370709.

[93] Maron B.J., Towbin J.A., Thiene G., Antzelevitch C., Corrado D., Arnett D., Moss A.J., Seidman C.E., Young J.B. (2006). Contemporary Definitions and Classification of the Cardiomyopathies. Circulation.

[94] Brieler J., Breeden M.A., Tucker J. (2017). Cardiomyopathy: An Overview. Am. Fam. Physician.

[95] Firth J. (2019). Cardiology: Hypertrophic cardiomyopathy. Clin. Med..

[96] Semsarian C., Ingles J., Maron M.S., Maron B.J. (2015). New Perspectives on the Prevalence of Hypertrophic Cardiomyopathy. J. Am. Coll. Cardiol..

[97] Maron B.J., Maron M.S. (2013). Hypertrophic cardiomyopathy. Lancet.

[98] James V., Nizamudeen Z.A., Lea D., Dottorini T., Holmes T.L., Johnson B.B., Arkell K.P., Denning C., Smith J.G.W. (2021). Transcriptomic Analysis of Cardiomyocyte Extracellular Vesicles in Hypertrophic Cardiomyopathy Reveals Differential snoRNA Cargo. Stem Cells Dev..

[99] Rizzuto A., Macchi C., Faggiano A., Calcagnino M., Baroni M., Gnan E., Paganini S., Giusti I., Dolo V., Corsini A. (2024). Extracellular vesicles characterization in patients with hypertrophic cardiomyopathy. Eur. Atheroscler. J..

[100] Tian C., Hu G., Gao L., Hackfort B.T., Zucker I.H. (2020). Extracellular vesicular MicroRNA-27a* contributes to cardiac hypertrophy in chronic heart failure. J. Mol. Cell. Cardiol..

[101] Bang C., Batkai S., Dangwal S., Gupta S.K., Foinquinos A., Holzmann A., Just A., Remke J., Zimmer K., Zeug A. (2014). Cardiac fibroblast–derived microRNA passenger strand-enriched exosomes mediate cardiomyocyte hypertrophy. J. Clin. Investig..

[102] Huang F., Na N., Ijichi T., Wu X., Miyamoto K., Ciullo A., Tran M., Li L., Ibrahim A., Marbán E. (2021). Exosomally derived Y RNA fragment alleviates hypertrophic cardiomyopathy in transgenic mice. Mol. Ther. Nucleic Acids.

[103] Mahmaljy H., Yelamanchili V.S., Singhal M. Dilated Cardiomyopathy. StatPearls [Internet]. https://www.ncbi.nlm.nih.gov/books/NBK441911/.

[104] Caño-Carrillo S., Castillo-Casas J.M., Franco D., Lozano-Velasco E. (2024). Unraveling the Signaling Dynamics of Small Extracellular Vesicles in Cardiac Diseases. Cells.

[105] Masarone D., Kaski J.P., Pacileo G., Elliott P.M., Bossone E., Day S.M., Limongelli G. (2018). Epidemiology and Clinical Aspects of Genetic Cardiomyopathies. Heart Fail. Clin..

[106] Zhang L., Zhang G., Lu Y., Gao J., Qin Z., Xu S., Wang Z., Xu Y., Yang Y., Zhang J. (2023). Differential expression profiles of plasma exosomal microRNAs in dilated cardiomyopathy with chronic heart failure. J. Cell. Mol. Med..

[107] Lin R., Rahtu-Korpela L., Szabo Z., Kemppi A., Skarp S., Kiviniemi A.M., Lepojärvi E.S., Halmetoja E., Kilpiö T., Porvari K. (2022). MiR-185-5p regulates the development of myocardial fibrosis. J. Mol. Cell. Cardiol..

[108] Surma S., Banach M. (2021). Fibrinogen and Atherosclerotic Cardiovascular Diseases-Review of the Literature and Clinical Studies. Int. J. Mol. Sci..

[109] Wolberg A.S. (2016). Primed to Understand Fibrinogen in Cardiovascular Disease. Arterioscler. Thromb. Vasc. Biol..

[110] Roura S., Gámez-Valero A., Lupón J., Gálvez-Montón C., Borràs F.E., Bayes-Genis A. (2018). Proteomic signature of circulating extracellular vesicles in dilated cardiomyopathy. Lab. Investig..

[111] Stack A.G., Mutwali A.I., Nguyen H.T., Cronin C.J., Casserly L.F., Ferguson J. (2014). Transferrin saturation ratio and risk of total and cardiovascular mortality in the general population. QJM Int. J. Med..

[112] Klip I.T., Comin-Colet J., Voors A.A., Ponikowski P., Enjuanes C., Banasiak W., Lok D.J., Rosentryt P., Anker A., Polonski L. (2013). Iron deficiency in chronic heart failure: An international pooled analysis. Am. Heart J..

[113] Enjuanes C., Klip I.T., Bruguera J., Cladellas M., Ponikowski P., Banasiak W., van Veldhuisen D.J., van der Meer P., Jankowska E.A., Comín-Colet J. (2014). Iron deficiency and health-related quality of life in chronic heart failure: Results from a multicenter European study. Int. J. Cardiol..

[114] Voss Winther S., Landt E., Nordestgaard B., Seersholm N., Dahl M. (2022). α1-Antitrypsin deficiency associated with increased risk of heart failure in two large populations. 0601—Epidemiology.

[115] Wu T., Chen Y., Du Y., Tao J., Zhou Z., Yang Z. (2018). Serum Exosomal MiR-92b-5p as a Potential Biomarker for Acute Heart Failure Caused by Dilated Cardiomyopathy. Cell. Physiol. Biochem..

[116] Sun X., Shan A., Wei Z., Xu B. (2018). Intravenous mesenchymal stem cell-derived exosomes ameliorate myocardial inflammation in the dilated cardiomyopathy. Biochem. Biophys. Res. Commun..

[117] Ni J., Liu Y., Wang K., Wu M., Kang L., Sha D., Xu B., Gu R. (2020). Trophoblast Stem-Cell-Derived Exosomes Improve Doxorubicin-Induced Dilated Cardiomyopathy by Modulating the let-7i/YAP Pathway. Mol. Ther. Nucleic Acids.

[118] Zhang W., Chen Z., Qiao S., Chen S., Zheng H., Wei X., Li Q., Xu B., Huang W. (2022). The effects of extracellular vesicles derived from Krüppel-Like Factor 2 overexpressing endothelial cells on the regulation of cardiac inflammation in the dilated cardiomyopathy. J. Nanobiotechnol..

[119] World Health Organization The Top 10 Causes of Death. https://www.who.int/news-room/fact-sheets/detail/the-top-10-causes-of-death.

[120] National Institute of Diabetes and Digestive and Kidney Diseases Diabetes. https://www.niddk.nih.gov/about-niddk/research-areas/diabetes.

[121] Yeung A.M., Huang J., Pandey A., Hashim I.A., Kerr D., Pop-Busui R., Rhee C.M., Shah V.N., Bally L., Bayes-Genis A. (2023). Biomarkers for the Diagnosis of Heart Failure in People with Diabetes: A Consensus Report from Diabetes Technology Society. Prog. Cardiovasc. Dis..

[122] Seferović P.M., Paulus W.J. (2015). Clinical diabetic cardiomyopathy: A two-faced disease with restrictive and dilated phenotypes. Eur. Heart J..

[123] Jia G., Whaley-Connell A., Sowers J.R. (2018). Diabetic cardiomyopathy: A hyperglycaemia- and insulin-resistance-induced heart disease. Diabetologia.

[124] de Gonzalo-Calvo D., van der Meer R.W., Rijzewijk L.J., Smit J.W.A., Revuelta-Lopez E., Nasarre L., Escola-Gil J.C., Lamb H.J., Llorente-Cortes V. (2017). Serum microRNA-1 and microRNA-133a levels reflect myocardial steatosis in uncomplicated type 2 diabetes. Sci. Rep..

[125] Huang J.P., Chang C.C., Kuo C.Y., Huang K.J., Sokal E.M., Chen K.H., Hung L.-M. (2022). Exosomal microRNAs miR-30d-5p and miR-126a-5p Are Associated with Heart Failure with Preserved Ejection Fraction in STZ-Induced Type 1 Diabetic Rats. Int. J. Mol. Sci..

[126] Watson C.J., Gupta S.K., O’Connell E., Thum S., Glezeva N., Fendrich J., Gallagher J., Ledwidge M., Grote-Levi L., McDonald K. (2015). MicroRNA signatures differentiate preserved from reduced ejection fraction heart failure. Eur. J. Heart Fail..

[127] Vegter E.L., van der Meer P., de Windt L.J., Pinto Y.M., Voors A.A. (2016). MicroRNAs in heart failure: From biomarker to target for therapy. Eur. J. Heart Fail..

[128] Li J., Salvador A.M., Li G., Valkov N., Ziegler O., Yeri A., Xiao C.Y., Mechcovetov B., Alsop E., Rodosthenous R.S. (2021). Mir-30d Regulates Cardiac Remodeling by Intracellular and Paracrine Signaling. Circ. Res..

[129] Zhang Y., Zhu Z., Wang T., Dong Y., Fan Y., Sun D. (2021). TGF-β1-containing exosomes from cardiac microvascular endothelial cells mediate cardiac fibroblast activation under high glucose conditions. Biochem. Cell Biol..

[130] Hu J., Wang S., Xiong Z., Cheng Z., Yang Z., Lin J., Wang T., Feng X., Gao E., Wang H. (2018). Exosomal Mst1 transfer from cardiac microvascular endothelial cells to cardiomyocytes deteriorates diabetic cardiomyopathy. Biochim. Biophys. Acta (BBA)—Mol. Basis Dis..

[131] Wang X., Gu H., Huang W., Peng J., Li Y., Yang L., Qin D., Essandoh K., Wang Y., Peng T. (2016). Hsp20-Mediated Activation of Exosome Biogenesis in Cardiomyocytes Improves Cardiac Function and Angiogenesis in Diabetic Mice. Diabetes.

[132] Lin Y., Zhang F., Lian X.-F., Peng W.-Q., Yin C.-Y. (2019). Mesenchymal stem cell-derived exosomes improve diabetes mellitus-induced myocardial injury and fibrosis via inhibition of TGF-Î^2^1/Smad2 signaling pathway. Cell. Mol. Biol..

[133] Zhen J., Bai J., Liu J., Men H., Yu H. (2024). Ginsenoside RG1-induced mesenchymal stem cells alleviate diabetic cardiomyopathy through secreting exosomal circNOTCH1 to promote macrophage M2 polarization. Phytother. Res..

[134] Nesheiwat Z., Goyal A., Jagtap M. (2024). Atrial Fibrillation.

[135] Kornej J., Börschel C.S., Benjamin E.J., Schnabel R.B. (2020). Epidemiology of Atrial Fibrillation in the 21st Century. Circ. Res..

[136] Wang S., Min J., Yu Y., Yin L., Wang Q., Shen H., Yang J., Zhang P., Xiao J., Wang Z. (2019). Differentially expressed miRNAs in circulating exosomes between atrial fibrillation and sinus rhythm. J. Thorac. Dis..

[137] Siwaponanan P., Kaewkumdee P., Phromawan W., Udompunturak S., Chomanee N., Udol K., Pattanapanasat K., Krittayaphong R. (2022). Increased expression of six-large extracellular vesicle-derived miRNAs signature for nonvalvular atrial fibrillation. J. Transl. Med..

[138] Zhu P., Li H., Zhang A., Li Z., Zhang Y., Ren M., Zhang Y., Hou Y. (2022). MicroRNAs sequencing of plasma exosomes derived from patients with atrial fibrillation: miR-124-3p promotes cardiac fibroblast activation and proliferation by regulating AXIN1. J. Physiol. Biochem..

[139] Wang S., Li L., Hu X., Liu T., Jiang W., Wu R., Ren Y., Wang M. (2021). Effects of Atrial Fibrillation-Derived Exosome Delivery of miR-107 to Human Umbilical Vein Endothelial Cells. DNA Cell Biol..

[140] Bai C., Liu Y., Zhao Y., Ye Q., Zhao C., Liu Y., Wang J. (2022). Circulating exosome-derived miR-122-5p is a novel biomarker for prediction of postoperative atrial fibrillation. J. Cardiovasc. Transl. Res..

[141] Liu D., Yang M., Yao Y., He S., Wang Y., Cao Z., Chen H., Fu Y., Liu H., Zhao Q. (2022). Cardiac Fibroblasts Promote Ferroptosis in Atrial Fibrillation by Secreting Exo-miR-23a-3p Targeting SLC7A11. Oxid. Med. Cell. Longev..

[142] Parent S., Vaka R., Risha Y., Ngo C., Kanda P., Nattel S., Khan S., Courtman D., Stewart D.J., Davis D.R. (2023). Prevention of atrial fibrillation after open-chest surgery with extracellular vesicle therapy. JCI Insight.

[143] Hao H., Yan S., Zhao X., Han X., Fang N., Zhang Y., Dai C., Li W., Yu H., Gao Y. (2022). Atrial myocyte-derived exosomal microRNA contributes to atrial fibrosis in atrial fibrillation. J. Transl. Med..

[144] Li S., Gao Y., Liu Y., Li J., Yang X., Hu R., Liu J., Zhang Y., Zuo K., Li K. (2020). Myofibroblast-Derived Exosomes Contribute to Development of a Susceptible Substrate for Atrial Fibrillation. Cardiology.

[145] Shaihov-Teper O., Ram E., Ballan N., Brzezinski R.Y., Naftali-Shani N., Masoud R., Ziv T., Lewis N., Schary Y., Levin-Kotler L.P. (2021). Extracellular Vesicles From Epicardial Fat Facilitate Atrial Fibrillation. Circulation.

[146] Cao F., Li Z., Ding W., Yan L., Zhao Q. (2021). Angiotensin II-Treated Cardiac Myocytes Regulate M1 Macrophage Polarization via Transferring Exosomal PVT1. J. Immunol. Res..

[147] Weiss L., Keaney J., Szklanna P.B., Prendiville T., Uhrig W., Wynne K., Kelliher S., Ewins K., Comer S.P., Egan K. (2021). Nonvalvular atrial fibrillation patients anticoagulated with rivaroxaban compared with warfarin exhibit reduced circulating extracellular vesicles with attenuated pro-inflammatory protein signatures. J. Thromb. Haemost..

[148] Li J., Zhang Q., Jiao H. (2021). LncRNA NRON promotes M2 macrophage polarization and alleviates atrial fibrosis through suppressing exosomal miR-23a derived from atrial myocytes. J. Formos. Med. Assoc..

[149] Xu L., Fan Y., Wu L., Zhang C., Chu M., Wang Y., Zhuang W. (2022). Exosomes from Bone Marrow Mesenchymal Stem Cells with Overexpressed Nrf2 Inhibit Cardiac Fibrosis in Rats with Atrial Fibrillation. Cardiovasc. Ther..

[150] Inamdar A.A., Inamdar A.C. (2016). Heart Failure: Diagnosis, Management and Utilization. J. Clin. Med..

[151] Schwinger R.H.G. (2021). Pathophysiology of heart failure. Cardiovasc. Diagn. Ther..

[152] Han C., Yang J., Sun J., Qin G. (2022). Extracellular vesicles in cardiovascular disease: Biological functions and therapeutic implications. Pharmacol. Ther..

[153] Matsumoto S., Sakata Y., Suna S., Nakatani D., Usami M., Hara M., Kitamura T., Hamasaki T., Nanto S., Kawahara Y. (2013). Circulating p53-Responsive MicroRNAs Are Predictive Indicators of Heart Failure After Acute Myocardial Infarction. Circ. Res..

[154] Xie Y., Hang J.Z., Zhang N., Liu G. (2022). Clinical Significance of MiR-27a Expression in Serum Exosomes in Patients with Heart Failure. Cell. Mol. Biol..

[155] Beg F., Wang R., Saeed Z., Devaraj S., Masoor K., Nakshatri H. (2017). Inflammation-associated microRNA changes in circulating exosomes of heart failure patients. BMC Res. Notes.

[156] Galluzzo A., Gallo S., Pardini B., Birolo G., Fariselli P., Boretto P., Vitacolonna A., Peraldo-Neia C., Spilinga M., Volpe A. (2021). Identification of novel circulating microRNAs in advanced heart failure by next-generation sequencing. ESC Heart Fail..

[157] Vilella-Figuerola A., Padró T., Roig E., Mirabet S., Badimon L. (2022). New factors in heart failure pathophysiology: Immunity cells release of extracellular vesicles. Front. Cardiovasc. Med..

[158] Verbree-Willemsen L., Zhang Y., Ibrahim I., Ooi S.B.S., Wang J., Mazlan M.I., Kuan W.S., Chan S.P., Peelen L.M., Grobbee D.E. (2020). Extracellular vesicle Cystatin C and CD14 are associated with both renal dysfunction and heart failure. ESC Heart Fail..

[159] Xiao Y.C., Wang W., Gao Y., Li W.Y., Tan X., Wang Y.K., Wang W.Z. (2022). The Peripheral Circulating Exosomal microRNAs Related to Central Inflammation in Chronic Heart Failure. J. Cardiovasc. Transl. Res..

[160] Tian C., Gao L., Zimmerman M.C., Zucker I.H. (2018). Myocardial infarction-induced microRNA-enriched exosomes contribute to cardiac Nrf2 dysregulation in chronic heart failure. Am. J. Physiol. Heart Circ. Physiol..

[161] Wang L., Liu J., Xu B., Liu Y.L., Liu Z. (2018). Reduced exosome miR-425 and miR-744 in the plasma represents the progression of fibrosis and heart failure. Kaohsiung J. Med. Sci..

[162] van den Hoogen P., de Jager S.C.A., Mol E.A., Schoneveld A.S., Huibers M.M.H., Vink A., Doevendans P.A., Laman J.D., Sluijter J.P.G. (2019). Potential of mesenchymal- and cardiac progenitor cells for therapeutic targeting of B-cells and antibody responses in end-stage heart failure. PLoS ONE.

[163] Ren Y., Wu Y., He W., Tian Y., Zhao X. (2023). Exosomes secreted from bone marrow mesenchymal stem cells suppress cardiomyocyte hypertrophy through Hippo-YAP pathway in heart failure. Genet. Mol. Biol..

[164] Xuan L., Fu D., Zhen D., Wei C., Bai D., Yu L., Gong G. (2022). Extracellular vesicles derived from human bone marrow mesenchymal stem cells protect rats against acute myocardial infarction-induced heart failure. Cell Tissue Res..

[165] Zhong Z., Tian Y., Luo X., Zou J., Wu L., Tian J. (2021). Extracellular Vesicles Derived From Human Umbilical Cord Mesenchymal Stem Cells Protect Against DOX-Induced Heart Failure Through the miR-100-5p/NOX4 Pathway. Front. Bioeng. Biotechnol..

[166] Duan J., Liu X., Shen S., Tan X., Wang Y., Wang L., Kang L., Wang K., Wei Z., Qi Y. (2023). Trophoblast Stem-Cell-Derived Exosomes Alleviate Cardiotoxicity of Doxorubicin via Improving Mfn2-Mediated Mitochondrial Fusion. Cardiovasc. Toxicol..

[167] Ni J., Liu Y., Kang L., Wang L., Han Z., Wang K., Xu B., Gu R. (2020). Human trophoblast-derived exosomes attenuate doxorubicin-induced cardiac injury by regulating miR-200b and downstream Zeb1. J. Nanobiotechnol..

[168] Ateeq M., Broadwin M., Sellke F.W., Abid M.R. (2024). Extracellular Vesicles’ Role in Angiogenesis and Altering Angiogenic Signaling. Med. Sci..

[169] Gnecchi M., He H., Noiseux N., Liang O.D., Zhang L., Morello F., Mu H., Melo L.G., Pratt R.E., Ingwall J.S. (2006). Evidence supporting paracrine hypothesis for Akt-modified mesenchymal stem cell-mediated cardiac protection and functional improvement. FASEB J..

[170] Potz B.A., Scrimgeour L.A., Pavlov V.I., Sodha N.R., Abid M.R., Sellke F.W. (2018). Extracellular Vesicle Injection Improves Myocardial Function and Increases Angiogenesis in a Swine Model of Chronic Ischemia. J. Am. Heart Assoc..

[171] Anderson J.D., Johansson H.J., Graham C.S., Vesterlund M., Pham M.T., Bramlett C.S., Montgomery E.N., Mellema M.S., Bardini R.L., Contreras Z. (2016). Comprehensive Proteomic Analysis of Mesenchymal Stem Cell Exosomes Reveals Modulation of Angiogenesis via Nuclear Factor-KappaB Signaling. Stem Cells.

[172] Shabbir A., Cox A., Rodriguez-Menocal L., Salgado M., Van Badiavas E. (2015). Mesenchymal Stem Cell Exosomes Induce Proliferation and Migration of Normal and Chronic Wound Fibroblasts, and Enhance Angiogenesis In Vitro. Stem Cells Dev..

[173] Todorova D., Simoncini S., Lacroix R., Sabatier F., Dignat-George F. (2017). Extracellular Vesicles in Angiogenesis. Circ. Res..

[174] Liang X., Zhang L., Wang S., Han Q., Zhao R.C. (2016). Exosomes secreted by mesenchymal stem cells promote endothelial cell angiogenesis by transferring miR-125a. J. Cell. Sci..

[175] Zhang B., Wu X., Zhang X., Sun Y., Yan Y., Shi H., Zhu Y., Wu L., Pan Z., Zhu W. (2015). Human Umbilical Cord Mesenchymal Stem Cell Exosomes Enhance Angiogenesis Through the Wnt4/β-Catenin Pathway. Stem Cells Transl. Med..

[176] Beltrami A.P., Barlucchi L., Torella D., Baker M., Limana F., Chimenti S., Kasahara H.K., Rota M., Musso E., Urbanek K. (2003). Adult Cardiac Stem Cells Are Multipotent and Support Myocardial Regeneration. Cell.

[177] Marote A., Teixeira F.G., Mendes-Pinheiro B., Salgado A.J. (2016). MSCs-Derived Exosomes: Cell-Secreted Nanovesicles with Regenerative Potential. Front. Pharmacol..

[178] Yu B., Zhang X., Li X. (2014). Exosomes derived from mesenchymal stem cells. Int. J. Mol. Sci..

[179] Joladarashi D., Kishore R. (2022). Mesenchymal Stromal Cell Exosomes in Cardiac Repair. Curr. Cardiol. Rep..

[180] Sun D., Zhuang X., Xiang X., Liu Y., Zhang S., Liu C., Barnes S., Grizzle W., Miller D., Zhang H.G. (2010). A Novel Nanoparticle Drug Delivery System: The Anti-inflammatory Activity of Curcumin Is Enhanced When Encapsulated in Exosomes. Mol. Ther..

[181] Danilushkina A.A., Emene C.C., Barlev N.A., Gomzikova M.O. (2023). Strategies for Engineering of Extracellular Vesicles. Int. J. Mol. Sci..

[182] Noronha Nde C., Mizukami A., Caliári-Oliveira C., Cominal J.G., Rocha J.L.M., Covas D.T., Swiech K., Malmegrim K.C.R. (2019). Priming approaches to improve the efficacy of mesenchymal stromal cell-based therapies. Stem Cell Res. Ther..

[183] Najar M., Krayem M., Merimi M., Burny A., Meuleman N., Bron D., Raicevic G., Lagneaux L. (2018). Insights into inflammatory priming of mesenchymal stromal cells: Functional biological impacts. Inflamm. Res..

[184] Zhou Y., Tsai T.L., Li W.J. (2017). Strategies to retain properties of bone marrow-derived mesenchymal stem cells ex vivo. Ann. N. Y Acad. Sci..

[185] François M., Romieu-Mourez R., Li M., Galipeau J. (2012). Human MSC Suppression Correlates with Cytokine Induction of Indoleamine 2,3-Dioxygenase and Bystander M2 Macrophage Differentiation. Mol. Ther..

[186] Rovira Gonzalez Y.I., Lynch P.J., Thompson E.E., Stultz B.G., Hursh D.A. (2016). In vitro cytokine licensing induces persistent permissive chromatin at the Indoleamine 2,3-dioxygenase promoter. Cytotherapy.

[187] Li B., Li C., Zhu M., Zhang Y., Du J., Xu Y., Liu B., Gao F., Liu H., Cai J. (2017). Hypoxia-Induced Mesenchymal Stromal Cells Exhibit an Enhanced Therapeutic Effect on Radiation-Induced Lung Injury in Mice due to an Increased Proliferation Potential and Enhanced Antioxidant Ability. Cell. Physiol. Biochem..

[188] Khan I., Ali A., Akhter M.A., Naeem N., Chotani M.A., Mustafa T., Salim A. (2016). Preconditioning of mesenchymal stem cells with 2,4-dinitrophenol improves cardiac function in infarcted rats. Life Sci..

[189] Liu X.B., Wang J.A., Ji X.Y., Yu S.P., Wei L. (2014). Preconditioning of bone marrow mesenchymal stem cells by prolyl hydroxylase inhibition enhances cell survival and angiogenesis in vitro and after transplantation into the ischemic heart of rats. Stem Cell Res. Ther..

[190] Rustad K.C., Wong V.W., Sorkin M., Glotzbach J.P., Major M.R., Rajadas J., Longaker M.T., Gurtner G.C. (2012). Enhancement of mesenchymal stem cell angiogenic capacity and stemness by a biomimetic hydrogel scaffold. Biomaterials.

[191] Meng Q., Man Z., Dai L., Huang H., Zhang X., Hu X., Shao Z., Zhu J., Zhang J., Fu X. (2015). A composite scaffold of MSC affinity peptide-modified demineralized bone matrix particles and chitosan hydrogel for cartilage regeneration. Sci. Rep..

[192] Huang Z., Nooeaid P., Kohl B., Roether J.A., Schubert D.W., Meier C., Boccaccini A.R., Godkin O., Ertel W., Arens S. (2015). Chondrogenesis of human bone marrow mesenchymal stromal cells in highly porous alginate-foams supplemented with chondroitin sulfate. Mater. Sci. Eng. C.

[193] Bhang S.H., Lee S., Shin J.Y., Lee T.J., Kim B.S. (2012). Transplantation of Cord Blood Mesenchymal Stem Cells as Spheroids Enhances Vascularization. Tissue Eng. Part A.

[194] Cheng N.C., Chen S.Y., Li J.R., Young T.H. (2013). Short-Term Spheroid Formation Enhances the Regenerative Capacity of Adipose-Derived Stem Cells by Promoting Stemness, Angiogenesis, and Chemotaxis. Stem Cells Transl. Med..

[195] Bartosh T.J., Ylöstalo J.H., Mohammadipoor A., Bazhanov N., Coble K., Claypool K., Lee R.H., Choi H., Prockop D.J. (2010). Aggregation of human mesenchymal stromal cells (MSCs) into 3D spheroids enhances their antiinflammatory properties. Proc. Natl. Acad. Sci. USA.

[196] Liu J., Zhu P., Song P., Xiong W., Chen H., Peng W., Wang S., Li S., Fu Z., Wang Y. (2015). Pretreatment of Adipose Derived Stem Cells with Curcumin Facilitates Myocardial Recovery via Antiapoptosis and Angiogenesis. Stem Cells Int..

[197] Nie W., Wu G., Zhong H., Xie H.Y. (2021). Membrane vesicles nanotheranostic systems: Sources, engineering methods, and challenges. Biomed. Mater..

[198] Mentkowski K.I., Snitzer J.D., Rusnak S., Lang J.K. (2018). Therapeutic Potential of Engineered Extracellular Vesicles. AAPS J..

[199] Nan W., Zhang C., Wang H., Chen H., Ji S. (2022). Direct Modification of Extracellular Vesicles and Its Applications for Cancer Therapy: A Mini-Review. Front. Chem..

[200] Nakase I., Futaki S. (2015). Combined treatment with a pH-sensitive fusogenic peptide and cationic lipids achieves enhanced cytosolic delivery of exosomes. Sci. Rep..

[201] Lee J., Lee H., Goh U., Kim J., Jeong M., Lee J., Park J.-H. (2016). Cellular Engineering with Membrane Fusogenic Liposomes to Produce Functionalized Extracellular Vesicles. ACS Appl. Mater. Interfaces.

[202] Zou J., Shi M., Liu X., Jin C., Xing X., Qiu L., Tan W. (2019). Aptamer-Functionalized Exosomes: Elucidating the Cellular Uptake Mechanism and the Potential for Cancer-Targeted Chemotherapy. Anal. Chem..

[203] Zhupanyn P., Ewe A., Büch T., Malek A., Rademacher P., Müller C., Reinert A., Jaimes Y., Aigner A. (2020). Extracellular vesicle (ECV)-modified polyethylenimine (PEI) complexes for enhanced siRNA delivery in vitro and in vivo. J. Control. Release.

[204] Haney M.J., Klyachko N.L., Zhao Y., Gupta R., Plotnikova E.G., He Z., Patel T.H., Piroyan A., Sokolsky M., Kabanov A.V. (2015). Exosomes as drug delivery vehicles for Parkinson’s disease therapy. J. Control. Release.

[205] Bose R.J.C., Uday Kumar S., Zeng Y., Afjei R., Robinson E., Lau K., Bermudez A., Habte F., Pitteri S.J., Sinclair R. (2018). Tumor Cell-Derived Extracellular Vesicle-Coated Nanocarriers: An Efficient Theranostic Platform for the Cancer-Specific Delivery of Anti-miR-21 and Imaging Agents. ACS Nano.

[206] Kim M.S., Haney M.J., Zhao Y., Mahajan V., Deygen I., Klyachko N.L., Linsko E., Piroyan A., Sokolsky M., Okolie O. (2016). Development of exosome-encapsulated paclitaxel to overcome MDR in cancer cells. Nanomedicine.

[207] Yan F., Zhong Z., Wang Y., Feng Y., Mei Z., Li H., Chen X., Cai L., Li C. (2020). Exosome-based biomimetic nanoparticles targeted to inflamed joints for enhanced treatment of rheumatoid arthritis. J. Nanobiotechnol..

[208] Fuhrmann G., Serio A., Mazo M., Nair R., Stevens M.M. (2015). Active loading into extracellular vesicles significantly improves the cellular uptake and photodynamic effect of porphyrins. J. Control. Release.

[209] Lamichhane T.N., Raiker R.S., Jay S.M. (2015). Exogenous DNA Loading into Extracellular Vesicles via Electroporation is Size-Dependent and Enables Limited Gene Delivery. Mol. Pharm..

[210] Yuan D., Zhao Y., Banks W.A., Bullock K.M., Haney M., Batrakova E., Kabanov A.V. (2017). Macrophage exosomes as natural nanocarriers for protein delivery to inflamed brain. Biomaterials.

[211] Zhan Q., Yi K., Qi H., Li S., Li X., Wang Q., Wang Y., Liu C., Qiu M., Yuan X. (2020). Engineering blood exosomes for tumor-targeting efficient gene/chemo combination therapy. Theranostics.

[212] Yang L., Han D., Zhan Q., Li X., Shan P., Hu Y., Ding H., Wang Y., Zhang L., Zhang Y. (2019). Blood TfR+ exosomes separated by a pH-responsive method deliver chemotherapeutics for tumor therapy. Theranostics.

[213] van Niel G., Carter D.R.F., Clayton A., Lambert D.W., Raposo G., Vader P. (2022). Challenges and directions in studying cell–cell communication by extracellular vesicles. Nat. Rev. Mol. Cell Biol..

[214] Li G., Chen T., Dahlman J., Eniola-Adefeso L., Ghiran I.C., Kurre P., Lam W.A., Lang J.K., Marbán E., Martín P. (2023). Current challenges and future directions for engineering extracellular vesicles for heart, lung, blood and sleep diseases. J. Extracell. Vesicles.

[215] Maroto R., Zhao Y., Jamaluddin M., Popov V.L., Wang H., Kalubowilage M., Zhang Y., Luisi J., Sun H., Culbertson C.T. (2017). Effects of storage temperature on airway exosome integrity for diagnostic and functional analyses. J. Extracell. Vesicles.

[216] Zeng Y., Qiu Y., Jiang W., Shen J., Yao X., He X., Li L., Fu B., Liu X. (2022). Biological Features of Extracellular Vesicles and Challenges. Front. Cell Dev. Biol..

